# Supplementary material for: Circadian and Circalunar Clock Interactions in a Marine Annelid
Source: Cell Rep. 2013 Oct 17;5(1):99–113. doi: 10.1016/j.celrep.2013.08.031 (PMC3913041; doi:10.1016/j.celrep.2013.08.031)
Supplement: Document S1. Article plus Supplemental Information [file mmc1.pdf]

Cell Reports
Article

Cell PRESS
Open ACCESS

Circadian and Circalunar Clock Interactions in a Marine Annelid

Juliane Zantke,[1,2] Tomoko Ishikawa-Fujiwara,[3] Enrique Arboleda,[1,5] Claudia Lohs,[1,6] Katharina Schipany,[1,7] Natalia Hallay,[1,2] Andrew D. Straw,[4] Takeshi Todo,[3] and Kristin Tessmar-Raible[1,2,*]
[1]Max F. Perutz Laboratories, University of Vienna, Dr. Bohr-Gasse 9/4, 1030 Vienna, Austria
[2]Research Platform "Marine Rhythms of Life," University of Vienna, Dr. Bohr-Gasse 9/4, 1030 Vienna, Austria
[3]Department of Radiation Biology and Medical Genetics, Graduate School of Medicine, Osaka University, B4, 2-2 Yamadaoka, Suita 565-0871, Japan
[4]Research Institute of Molecular Pathology, University of Vienna, Dr. Bohr-Gasse 7, 1030 Vienna, Austria
[5]Present address: CIEE Research Station Bonaire, Kaya Gobernador Debrot 26, Kralendijk, Bonaire, the Netherlands
[6]Present address: Helmholtz Zentrum München Institute of Molecular Immunology, Marchioninistrasse 25, 81377 Munich, Germany
[7]Present address: Institute of Medical Genetics, Medical University of Vienna, Währinger Strasse 10, 1090 Vienna, Austria
*Correspondence: kristin.tessmar@mfpl.ac.at
http://dx.doi.org/10.1016/j.celrep.2013.08.031
This is an open-access article distributed under the terms of the Creative Commons Attribution License, which permits unrestricted use, distribution, and reproduction in any medium, provided the original author and source are credited.

SUMMARY

Life is controlled by multiple rhythms. Although the interaction of the daily (circadian) clock with environmental stimuli, such as light, is well documented, its relationship to endogenous clocks with other periods is little understood. We establish that the marine worm *Platynereis dumerilii* possesses endogenous circadian and circalunar (monthly) clocks and characterize their interactions. The RNAs of likely core circadian oscillator genes localize to a distinct nucleus of the worm's forebrain. The worm's forebrain also harbors a circalunar clock entrained by nocturnal light. This monthly clock regulates maturation and persists even when circadian clock oscillations are disrupted by the inhibition of casein kinase 1δ/ε. Both circadian and circalunar clocks converge on the regulation of transcript levels. Furthermore, the circalunar clock changes the period and power of circadian behavior, although the period length of the daily transcriptional oscillations remains unaltered. We conclude that a second endogenous noncircadian clock can influence circadian clock function.

INTRODUCTION

Most, if not all, organisms feed periodic changes in light conditions into molecular clockworks that allow them to anticipate rhythmic changes in their environment and to synchronize their behavior and physiology (Cold Spring Harbor Symposia on Quantitative Biology, 2007; Roenneberg and Merrow, 2005). Efforts to study the underlying molecular mechanisms have focused almost exclusively on circadian clocks (i.e., clocks anticipating daily cycles). One of the critical mechanisms driving animal circadian clocks are transcriptional/translational feedback loops formed by a set of regulatory genes. These genes are partially shared between insect and mammalian models, arguing for a common origin of animal circadian clocks. The feedback loops continue to run under constant conditions and are coordinated with the animal's environment by entrainment (Cold Spring Harbor Symposia on Quantitative Biology, 2007; Roenneberg and Merrow, 2005).

However, many organisms also exhibit rhythms of longer and shorter period lengths (Aschoff, 1981; Naylor, 2010). In order to maximize the chance of finding mature mates, to avoid predators, and to have favorable environmental conditions, organisms ranging from brown algae and corals to worms and vertebrates synchronize their maturation and spawning to a particular moon phase, to particular times of the day, and/or to specific seasons within a year (Fox, 1924; Harrison et al., 1984; Korringa, 1947; Tessmar-Raible et al., 2011). As with circadian rhythms, such noncircadian (e.g., annual and monthly) rhythms are often driven by internal oscillators (circannual and circalunar clocks, respectively), which use light cues (photoperiod and moonlight, respectively) for the adjustment with the outer environmental conditions (Dupré and Loudon, 2007; Franke, 1985; Lincoln et al., 2006; Naylor, 2010; Hazlerigg and Lincoln, 2011; Kaiser et al., 2011).

Numerous studies have assessed the influence of additional light cues on the molecules and function of the circadian clock. Photoperiod influences circadian clock gene oscillations in insects (Koštál, 2011) and the waveform of circadian oscillations in mice (Ciarleglio et al., 2011), resulting in activity differences between animals raised under different photoperiods. Likewise, dim nocturnal light at moonlight intensity has been shown to influence circadian clock gene expression levels and timing in *Drosophila*, resulting in elevated nocturnal activity under laboratory conditions (Bachleitner et al., 2007). However, no elevated nocturnal activity was observed in corresponding moon phases under natural conditions, and the level of Period was differently altered (Vanin et al., 2012). Lunar light influences the levels of the putative light receptor and/or core clock gene *cryptochrome* in the circalunar spawning coral *Acropora* and the rabbit fish

CrossMark

Siganus guttatus (Fukushiro et al., 2011; Levy et al., 2007). In *Siganus*, moonlight has also been shown to elevate levels of the circadian clock gene *per2* (Sugama et al., 2008). However, a key issue that has remained obscure is if and how circadian and noncircadian internal oscillators interact molecularly to influence the behavior of an organism, independent of illumination effects.

A suitable model system to assess this question has to be a molecularly accessible, extant animal that at the same time possesses circadian and noncircadian timing mechanisms. The bristle worm *Platynereis dumerilii* offers this dual advantage. *Platynereis* was among the first species for which a circalunar spawning rhythm was scientifically documented (Fage and Legendre, 1927; Ranzi, 1931a, 1931b). In addition, *Platynereis* has emerged as a highly suitable model for molecular neurobiology (Arendt et al., 2004; Backfisch et al., 2013; Tessmar-Raible et al., 2007; Tomer et al., 2010).

Here, we establish that *Platynereis dumerilii* possesses both a circadian and a circalunar clock. Whereas the circalunar-clock-controlled reproductive timing rhythms are insensitive to the functional disruption of circadian clock gene oscillations, the circalunar clock affects the circadian clock on at least two levels. First, the period length and power of circadian-clock-controlled locomotor behaviors are significantly different between different phases of the circalunar clock, while the period length of the presumptive core circadian clock molecular oscillations remains unaffected. Second, *clock*, *period*, *pdp1*, and *timeless* transcript levels oscillate in specific brain nuclei of the worm's forebrain not only over 24 hr, but also across different phases of the lunar month. This establishes changes in RNA levels as a direct or indirect output of the circalunar clock.

RESULTS

Platynereis Possesses a Light-Entrained Circalunar Clock

The circalunar reproductive periodicity of *Platynereis dumerilii* (Figures 1A and 1B) has been extensively documented (Figure S1). Reproductive state, as measured by the number of animals reaching sexual maturity, is maximal shortly after new moon (NM) and minimal during periods of full moon (FM) (Figure S1).

We first assessed if our *Platynereis dumerilii* culture also possesses a nocturnal-light-adjusted circalunar spawning cycle. Following the conditions used in classical experiments (Hauenschild, 1954, 1955, 1960), we subjected the culture to a circadian light regimen of 16 hr light and 8 hr darkness (Figure 1C). For eight consecutive nights of a lunar month, we exposed the worms to dim nocturnal light (termed "full moon period" [FM]). We refer to the middle week of the remaining period as NM. This monthly light cycle in the lab can be in phase (Figure 1D) or out of phase (Figure 1E) with the natural moon. In accordance with classical observations (Figure S1), the daily number of mature animals peaked at the time between the FM stimuli (Figures 1D and 1E). Irrespective of the natural moon phase, these peaks of maturing animals remained in phase with respect to the week of the nocturnal light stimulus (Figures 1D and 1E). This establishes that nocturnal light stimuli alone are sufficient to synchronize circalunar reproductive cycles in our *Platynereis* lab culture.

Next, we tested if the observed circalunar spawning rhythm was controlled by an endogenous circalunar clock. As this point was debated previously (Hauenschild, 1960; Palmer, 1974), we performed lunar free-running experiments. After entrainment of animals for more than 2 months in the described circadian and circalunar light regimens, the FM stimulus was omitted, whereas the circadian light cycle remained unchanged (termed "free-running full moon" [FR-FM] in Figure 1C). The NM after this FR-FM is termed "free-running new moon" (FR-NM in Figure 1C). Worms continued to exhibit a monthly reproductive periodicity under these conditions (Figure 1F), with a period of 30 days (Figure 1G). Worms under constant light or raised without any nocturnal illumination did not show reproductive rhythms (Figure 1H). This establishes that circalunar reproductive periodicity in our culture is governed by an endogenous circalunar clock.

Platynereis Possesses the Full Complement of *Drosophila* and Mouse Core Circadian Oscillator Gene Orthologs

After we established that our worms possessed an endogenous circalunar clock, we tested for the presence of an endogenous circadian clock. For this, we determined the worms' complement of core circadian clock genes and their expression dynamics. *Bmal*, *period*, and *clock* are present in the core circadian oscillator in vertebrates and flies (Young and Kay, 2001); *cryptchrome* (*cry*) acts as core clock component in vertebrates and nondrosophilid invertebrates (Chaves et al., 2011; Zhan et al., 2011; Zhu et al., 2005); *timeless* is crucial for the insect circadian clock (Myers et al., 1995), but the gene is absent from vertebrates (Gotter, 2006). Orthologs of *timeout* (also termed *tim2*) and *cry* are important for circadian clock entrainment in insects (Benna et al., 2010; Emery et al., 2000). Moreover, *cry* is part of the circadian oscillator in the fly peripheral clock (Ivanchenko et al., 2001; Krishnan et al., 2001; Levine et al., 2002). *Pdp1* acts together with *vrille* in a modulatory feedback loop on the core transcription/translational feedback loop in insects (Blau and Young, 1999; Cyran et al., 2003; Glossop et al., 2003). Of these genes, only *bmal* had been identified in larval *Platynereis* (Arendt et al., 2004). By a combination of degenerated PCR and massive transcript sequencing, we identified *Platynereis* orthologs of *period*, *clock*, *timeless*, *timeout*, *pdp1*, and *vrille*, as well as two *cry* genes that we name *L-cry* ("L" indicating orthology to light-receptive Crys) and *tr-cry* ("tr" indicating orthology to Crys acting as transcriptional repressors) (Figure S2A).

As transcriptional oscillations are important for circadian clock function (Kadener et al., 2008; Padmanabhan et al., 2012), we next investigated messenger RNA (mRNA) dynamics of these genes. In addition, we focused our analyses on premature adult heads (Figure 1A). It had previously been shown that the maturation of *Platynereis*, which is the major event known to be synchronized by circadian and circalunar clocks, is controlled by the brain (Hauenschild, 1964, 1966; Hofmann, 1975). In order to ensure that any observed changes were due to the experimental conditions, but not due to developmental stage differences of the worms, we carefully staged the worms based on segment numbers, appendage shape, pigment appearance, and eye and body size.

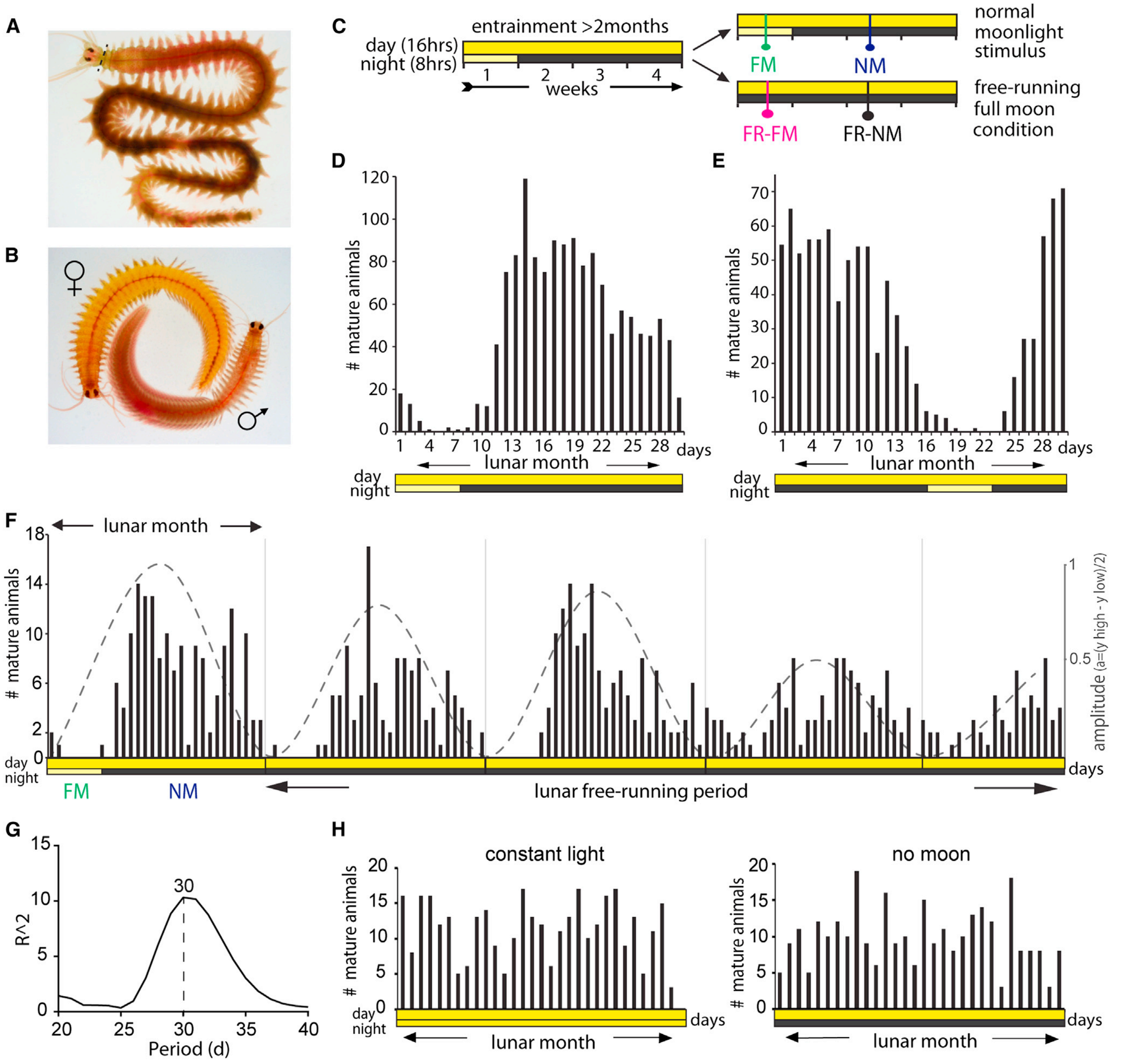

Figure 1. Circalunar Reproductive Periodicity of *Platynereis dumerilii* Is Entrained by Light and Controlled by a Clock Mechanism

(A) Premature adult (>2 months of age) as used in subsequent molecular and behavioral experiments is shown.

(B) Mature male and female as counted for the quantification of mature worms during mating dance are shown.

(C) Schematization of illumination conditions is shown. Daylight, yellow bars; nights without moon (new moon [NM]), black bars; nights with dim light simulating full moon (FM), light yellow bar. For “lunar” free-running experiments, the dim nocturnal light signal is omitted (FR-FM, free-running full moon; FR-NM, free running new moon). Illumination conditions used on x axis encode for 1; number of days, 2; day/night (in vertical direction).

(D and E) Light-entrained lab cultures exhibit maturation peaks comparable to nature (Figure S1). Nocturnal illumination in phase (D) and out of phase (E) with the natural moon is shown.

(F) Maturation synchronization continues for several months under circalunar free-running conditions after entrainment with dim nocturnal light (see C); dashed line indicates decreasing amplitude.

(G) Fourier analysis of free-running full moon spawning data shown in (F) reveals a 30-day period length, corresponding to the length of one lunar month.

(H) Worms grown under constant light (same light intensity during day/night) or without nocturnal illumination show no synchronization in maturation.

We first investigated the temporal expression profiles of *bmal*, *period*, *clock, tr-cry*, *timeless*, *vrille*, *pdp1*, and *timeout* using quantitative PCR (qPCR). This would also allow us to obtain an understanding of how the different circadian clock genes might relate to each other in terms of their regulation. We sampled heads during different circadian points at the

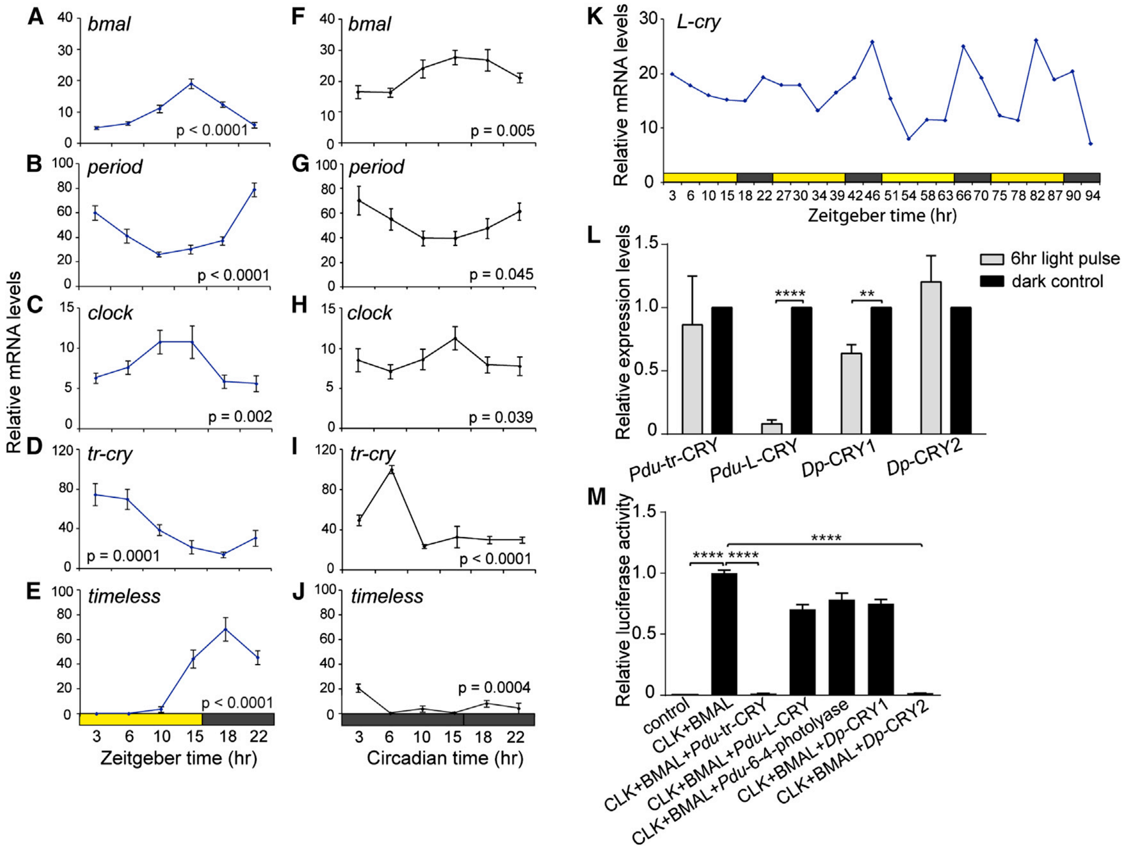

Figure 2. *Platynereis* Circadian Clock Gene Orthologs Show Circadian Oscillations on the RNA Level

(A–J) Temporal profiles of clock gene RNA expression in *Platynereis* heads sampled under NM (A–E) circadian light regimen and constant darkness (F–J) are shown. Values are means ± SEM, n = 5–16 (A–E), n = 6 (F–J); four to five heads/n. The p value was determined by one-way ANOVA. See Figures S2B–S2G for additional circadian clock genes.

(K) *Platynereis L-cry* transcript levels fluctuate, but do not show regular cycling patterns over 4 days (n = 2).

(L) Light decreases *Pdu*-L-Cry, but not *Pdu*-tr-Cry, levels in S2 cells. *Dp*-Cry1 and *Dp*-Cry2 serve as positive and negative controls, respectively. V5 epitope-tagged *Pdu*-L-Cry, *Pdu*-tr-Cry, *Dp*-Cry1, *Dp*-Cry2 was coexpressed with GFP. After a 6 hr light pulse (gray bars) or constant darkness (black bars), cell extracts were collected, western blotted, and probed with anti-V5 and anti-GFP (see Figure S2H). CRY levels were quantified by densitometry of antibody staining after normalization with GFP. The dark value for each CRY was plotted as 100%. Data are means ± SEM; n = 3 independent transfections. Significant differences were assessed by Student's t test (**$p < 0.01$; ****$p < 0.0001$).

(M) *Platynereis* tr-Cry, but not the closely related Pdu-L-Cry or Pdu-6-4-photolyase, strongly inhibit *Pdu*-CLK:*Pdu*-BMAL-mediated transcription in a luciferase reporter gene assays. The monarch butterfly *per* E-box-containing enhancer (*DpPer4Ep-Luc*) was used in the absence (control) or presence of *Pdu*-clock/*Pdu*-*bmal* plasmids (350 ng each). *Dp-cry1* and *Dp-cry2* serve as positive and negative controls, respectively. Data are means ± SEM; n = 4–8 independent transfections. Significant differences were determined by Student's t test (****$p < 0.0001$).

NM phase under light-dark (LD) conditions. In these experiments, *bmal*, *period*, *clock*, *tr-cry*, and *timeless* (Figures 2A–2E) and *vrille*, *pdp1*, and *timeout* (Figures S2B–S2D) exhibited robust circadian cycles. With the exception of *timeless* and *timeout*, this cycling was maintained during constant darkness (DD) (Figures 2F–2J; Figures S2E–S2G), consistent with the notion that *bmal*, *period*, *clock*, *pdp1*, *vrille*, and *tr-cry* are components of a core circadian oscillator in *Platynereis* heads. *Clock* and *bmal* transcripts cycled in phase with each other (Figures 2A, 2C, 2F, and 2H), consistent with a possible heterodimer formation known from flies to mammals (Darlington et al., 1998; Gekakis et al., 1998). *Period*, *pdp1*, and *timeout* transcript oscillations (Figures 2B and 2G; Figures S2C, S2D, S2F, and S2G) were in antiphase with *bmal/clock* expression. In contrast to *Drosophila*, where *vrille* RNA levels peak prior to *pdp1* levels (Cyran et al., 2003), *vrille* RNA peaks followed those of *pdp1* in *Platynereis* (Figures S2B, S2C, S2E, and S2F). *tr-cry* and *timeless* RNA level changes were neither directly in phase nor directly in antiphase with *bmal* and *clock*. They showed either high levels in the morning or during the evening/night (Figures 2D, 2E, 2I, and 2J). Furthermore, *timeless* transcripts displayed significantly lower levels under DD, as well as less pronounced and strongly phase-shifted circadian oscillations (Figures 2E and 2J), suggesting that the changes in its RNA level are predominantly directly controlled by light. Finally, transcriptional fluctuations of *L-cry* did not follow a clear circadian periodicity (Figure 2K).

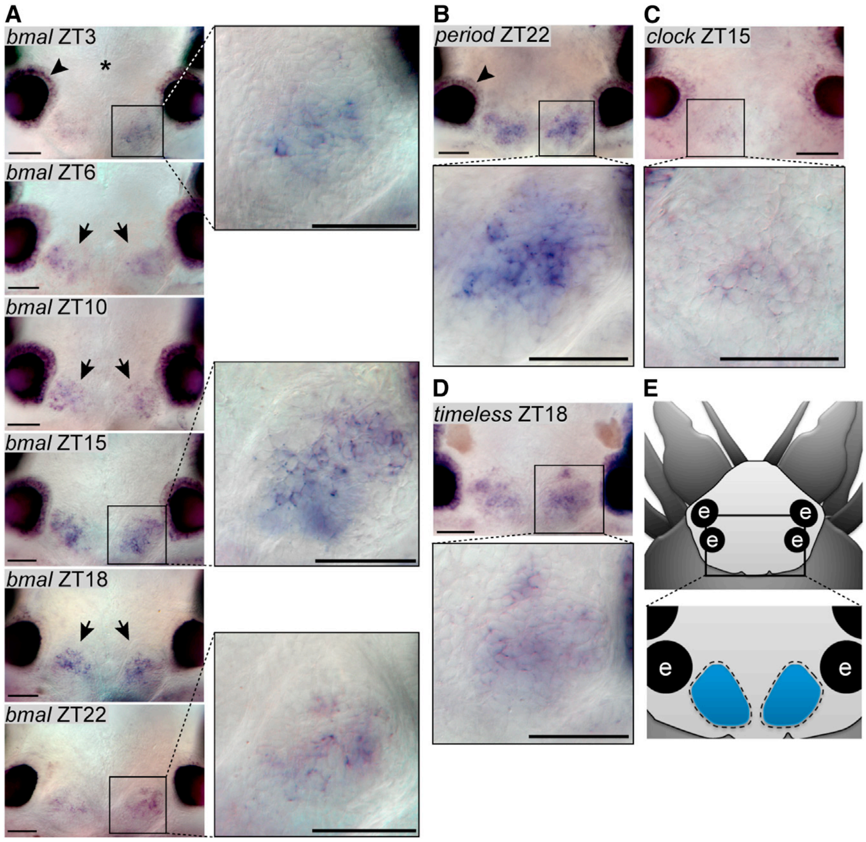

Figure 3. *Platynereis* Circadian Clock Gene Orthologs Are Confined to a Specific Brain Nucleus

(A–D) Whole-mount in situ hybridization of circadian clock genes on premature adult *Platynereis* heads is shown. Arrows point at the morphologically visible border of the medial brain nuclei expressing the genes. See also magnified view as indicated by the box; dorsal view, anterior to the top. For additional circadian clock genes, sense controls and expression of nonclock genes, see Figures S3A–S3F. Arrowheads indicate expression in eyes. Scale bar represents 50 μm, and asterisk indicates the position of major brain neuropil.

(E) Scheme of worm head indicating area is shown. Circadian clock gene expressing brain nuclei are indicated as blue ovals. e, adult eyes.

Pdu-L-Cryptochrome and *Pdu*-tr-Cryptochrome Can Function as a Light Receptor and Transcriptional Repressor, Respectively

In order to test if the investigated *Platynereis* circadian clock genes can indeed function in the conventional clock mechanism, we employed two assays previously used to validate the activity of presumptive core circadian clock genes of the monarch butterfly (Zhu et al., 2005, 2008). Cryptochromes functioning as light receptors undergo a light-dependent reduction in protein levels in S2 cells because of proteasome-mediated degradation (Lin et al., 2001; Zhu et al., 2005). If *Pdu*-L-CRY can indeed function as light receptor, we should be able to observe such a reduction. We assessed the effect of a 6 hr light pulse to promote *Pdu*-L-CRY, as well as *Pdu*-tr-CRY degradation, and compared the responses to those of the two monarch butterfly Cryptochrome proteins as positive and negative controls, respectively. We found that the *Platynereis* L-Cryptochrome, most closely related to *Dp*-Cry1 and *d*Cry, was strongly degraded under a 6 hr light pulse, whereas *Pdu*-tr-Cry was not affected (Figure 2L; Figure S2H). This suggests that *Pdu*-L-Cry can function as a light receptor, like its orthologs in the fruit fly and the monarch butterfly.

We further asked if *Pdu-bmal* and *Pdu-clock* are able to activate transcription from an E-box-containing construct. We constructed a luciferase construct, based on previous work in the monarch (Zhu et al., 2005; Yuan et al., 2007), containing two consensus E-boxes of the 5′ flanking region of *Dp-per*. Cotransfection of this construct with *Pdu-bmal* and *Pdu-clock* into S2 cells led to a strong activation of luciferase activity (Figure 2M). Additional transfection of *Pdu-tr-cry* strongly and highly significantly reduced this activation, similar to our positive control, the monarch butterfly's *tr-cry* ortholog, *cry2* (Figure 2M). Addition of *Pdu-L-cry* or its monarch ortholog *cry1* did not reduce *Pdu*-Bmal/*Pdu*-Clock-mediated luciferase expression in comparable levels (Figure 2M). Similarly, *Pdu-6-4photolyase,* a gene most closely related to *Pdu-tr-cry*, but whose orthologs function in UV-induced DNA repair (Sancar, 2008), did not show obvious transcriptional repressor activity (Figure 2M). We thus conclude that *bmal*, *clock*, and *tr-cry* likely function in a core circadian clock positive/negative transcriptional loop in *Platynereis dumerilii*, like their orthologs in other species.

Platynereis Core Circadian Clock Gene Orthologs Are Confined to Specific Domains in the Medial Forebrain

In order to determine if the uncovered circadian clock gene orthologs localize to a centralized structure or are broadly expressed, we performed whole-mount in situ hybridizations (WMISH).

All genes tested were specifically expressed in the posterior medial brain (Figures 3A–3E; Figures S3A–S3C), particularly in paired oval-shaped structures (arrows and magnifications in Figures 3A-3D; Figure S3A; compare Figures S3D and S3E for sense controls and Figure S3F for expression examples of two noncircadian transcription factors). These brain regions were already noted by Retzius as distinct nuclei in the brain of *Nereis*, a close relative of *Platynereis* (Retzius, 1895), hence representing nuclei conserved among nereidid worms. The brain morphology of *Platynereis* changes little during development from larvae to premature adults (Tomer et al., 2010). By position and relation to the axonal scaffold and the prominent cilia of the ciliary photoreceptor cells (arrows in Figures S3G–S3J), these distinct nuclei arise from the area demarcated by *bmal* in the 2-day-old larval

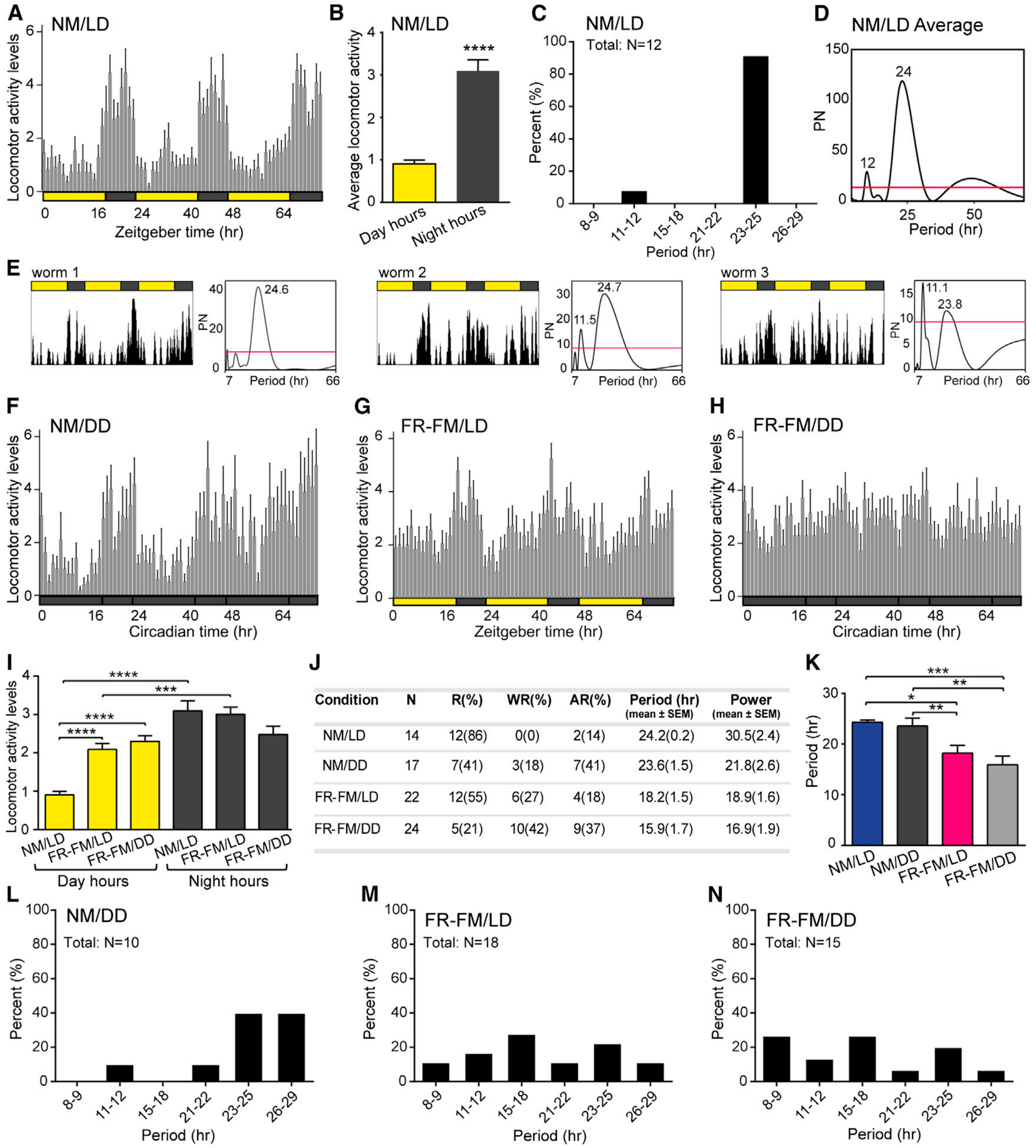

Condition	N	R(%)	WR(%)	AR(%)	Period (hr) (mean ± SEM)	Power (mean ± SEM)
NM/LD	14	12(86)	0(0)	2(14)	24.2(0.2)	30.5(2.4)
NM/DD	17	7(41)	3(18)	7(41)	23.6(1.5)	21.8(2.6)
FR-FM/LD	22	12(55)	6(27)	4(18)	18.2(1.5)	18.9(1.6)
FR-FM/DD	24	5(21)	10(42)	9(37)	15.9(1.7)	16.9(1.9)

Figure 4. The Circalunar Clock Affects Circadian-Clock-Controlled Activity Rhythms

(A) Mean locomotor activity (hourly average ± SEM) shows higher nocturnal activity in *Platynereis* in NM under 16:8LD circadian illumination over the course of 3 days (N = 12 rhythmic animals). Active behaviors were counted as 1, inactive as 0. See Figures S4A–S4C for details on active versus inactive behaviors and recoding setup.

(B) Quantification of average locomotor activity per hour of day hours (yellow bar) versus night hours (black bar) of 3 consecutive days is shown. Error bars represent ±SEM. Significant differences were determined by Student's t test (****$p < 0.0001$).

(C) Percentage of present period length of individual worms under NM/LD conditions is shown. See individual periodograms in Figure S4J.

(D) Average periodogram (N = 12) for NM/LD conditions shows a dominant period of 24 hr and an additional 12 hr peak. The red line indicates the significant p level = 0.05.

(E) Actograms and their corresponding periodogram of 3 individual worms recorded under NM/LD conditions are shown.

(F) *Platynereis* locomotor activity cycles continue in NM under complete darkness (DD) over at least 3 consecutive days (N = 10 rhythmic animals) showing a higher nocturnal activity. NM/DD: worms were entrained normally with circadian and circalunar illumination conditions. Recordings were performed during NM in complete darkness. See (A) for scoring details and Figure S4E for activity cycles including arrhythmic animals and Figures S4H and S4K for periodogram analysis.

(G) Mean locomotor activity cycles continue in FR-FM under normal light/dark (LD) conditions showing an increase in daily locomotor activity (N = 18 rhythmic animals). See (A) for scoring details and Figure 1C for details on illumination. See Figures S4F and S4L for activity cycles including arrhythmic animals and periodogram analysis, respectively.

(legend continued on next page)

medial forebrain (Arendt et al., 2004). Our findings of a medial forebrain nucleus harboring the core circadian clock genes are hence also consistent with our previous analyses in *Platynereis* larvae (Arendt et al., 2004).

The observed coexpression of the *Platynereis* clock gene orthologs is consistent with them acting together in a positive-negative feedback loop, as typical for the core circadian oscillators of all animals analyzed to date. Likewise, the expression of *L-cry* in the same oval-shaped posterior medial forebrain domains (Figure S3B; compare to Figures 3A–3E), along with the presented functional data, are consistent with L-Cry serving as a possible light sensor for the *Platynereis* circadian clock. This is also coherent with the fact that light should be able to reach these cells, as the worm's brain is relatively small and the cuticle largely transparent.

In addition to these nuclei, we also noted circadian clock gene expression in the area of the eyes (arrowhead in Figures 3A and 3B). Again, this staining was not present in sense controls, nor was it typically present when other genes were stained (Figures S3D–S3F). In order to analyze the exact position and extent of this expression, we performed WMISH on a *Platynereis* eye pigment mutant (Fischer, 1969). As in *Drosophila* (Hunter-Ensor et al., 1996), cells in the eyes also exhibited circadian clock gene expression (Figure S3K), albeit in general less than in the posterior medial forebrain domain.

We next analyzed if our WMISH confirms the daily transcriptional oscillations observed by qPCR. For this, we focused on the two most strongly expressed clock gene orthologs, *Pdu-bmal* and *Pdu-period*. When analyzed at different circadian time points, the expression of both genes showed circadian fluctuations within the described two medial brain nuclei (Figure 3A; Figure S3A), suggesting that these are the major circadian clock centers of *Platynereis*.

Platynereis Locomotor Activity Is under Circadian Clock Control

Given that *Platynereis* exhibits molecular circadian oscillations in paired medial forebrain nuclei, we next asked if the worms also displayed circadian behavior. We therefore recorded worms in a box over several days using an infrared camera and categorized their behavior into active (searching, fighting) and inactive (no movements, undulatory fanning movements) types (Figures S4A–S4C). We first analyzed if the worms showed any consistent activity patterns over multiples of 24 hr during NM/LD. The activity data were analyzed using ActogramJ for chronobiological analyses for rhythmicity and period lengths (Schmid et al., 2011). Under NM/LD conditions, the worms displayed primarily nocturnal activity (Figures 4A and 4B) with an average period length of 24.2 hr (±0.2) (Figures 4C-4E and 4J; Figures S4D and S4J). These data are consistent with the fact that the nuptial dance of *Platynereis* only occurs during few hours of the night (Korringa, 1947), and with previous observations in the related nereidid *Nereis* (Last, 2003; Last and Olive, 2004).

In addition, a dominant ~12 hr period was observed in 8% of the analyzed individuals (n = 14, Figures 4C and 4E). This 12 hr activity rhythm does not appear to be crepuscular, possibly rather resembling a circatidal rhythm (see example worm 3 in Figure 4E). Under NM/DD conditions, the worms continued to show a circadian periodicity (23.6 ± 1.5 hr) over at least 3 days (Figures 4F, 4J, and 4L; Figures S4E, S4H, and S4K), evidencing that the worm's locomotor activity is under circadian clock control. The generally still relatively high level of variability in the period lengths of our periodogram analyses might be due to the representative, yet still relatively short analyses timeframe and small sample size.

The Circalunar Clock Impacts Circadian Behavior

Having established that both circadian and circalunar clocks exist in *Platynereis*, we next investigated how these two clocks interact with each other.

We started by comparing the *Platynereis* circadian locomotor activity cycles between two different phases of the circalunar clock (NM versus FR-FM; see Figure 1C). Compared to NM, the worms were less rhythmic in their locomotor behavior under FR-FM in both circadian LD and DD conditions (Figures 4F–4H and 4J). Their activity during the day significantly increased (Figures 4G–4I), while the average period length significantly shortened to 18.2 hr (±1.5 hr) for FR-FM/LD and 15.9 hr (±1.7 hr) for FR-FM/DD, the power of the rhythm decreased to 18.9 (±1.6) and 16.9 (±1.9), respectively (Figures 4J and 4K; Figures S4F, S4G, and S4I). Analyses of the periodograms of individual animals revealed that such shorter period length occurred indeed on individual bases, but can vary from 8 hr to 18 hr (Figures 4M and 4N; Figures S4L and S4M). Occasionally, worms already showed rhythms of shorter period lengths during NM (LD and DD) (Figures 4C and 4L; Figures S4J and S4K). However, the

(H) *Platynereis* daily locomotor activity in FR-FM under complete darkness (DD) is flattened and displays a shorter period of about 18 hr (N = 15 rhythmic animals). See Figures S4G and S4M for activity cycles including arrhythmic animals and periodogram analysis, respectively.

(I) Quantification of average locomotor activity per hour of day hours (yellow bar) versus night hours (black bar) comparing NM/LD versus FR-FM/LD versus FR-FM/DD is shown. Worms under FR-FM/LD are nocturnal, but exhibit higher daily locomotor activity than during NM/LD. Worms in FR-FM under complete darkness (DD) show no nocturnal activity anymore, but an increase in daily activity. Error bars represent ±SEM. Significant differences were determined by Student's t test (***$p < 0.001$; ****$p < 0.0001$).

(J) Summary of Lomb-Scargle periodogram analyses of time series of locomotor activity observed under different circadian and circalunar conditions over the course of 3 days (see Figure 1C) is shown. Period and Power were calculated for all rhythmic worms. N, number of worms analyzed; R, rhythmic; WR, weakly rhythmic; AR, arrhythmic; see Experimental Procedures for classification. Data from three independent NM, DD, FR-FM experiments and from two independent FR-FM/DD were pooled, respectively.

(K) Worms in NM versus FR-FM show significant differences in circadian activity period length. Error bars represent ±SEM. Significant differences were determined by Student's t test (*$p < 0.05$; **$p < 0.01$; ***$p < 0.001$).

(L–N) Percentage of present period lengths of individual worms is shown. (L) Under the NM/DD condition, the circadian period is reduced to 40%. Worms show additional longer and shorter periods. (M) Under the FR-FM/LD condition, worms display additional periods of about 9 hr and 18 hr, which are not present in NM under LD or DD (compare C and L). (N) In the FR-FM under DD condition, worms show an increase in period lengths of about 18 hr and 9 hr, decreasing the percentage of other periods (compare C, L, and M).

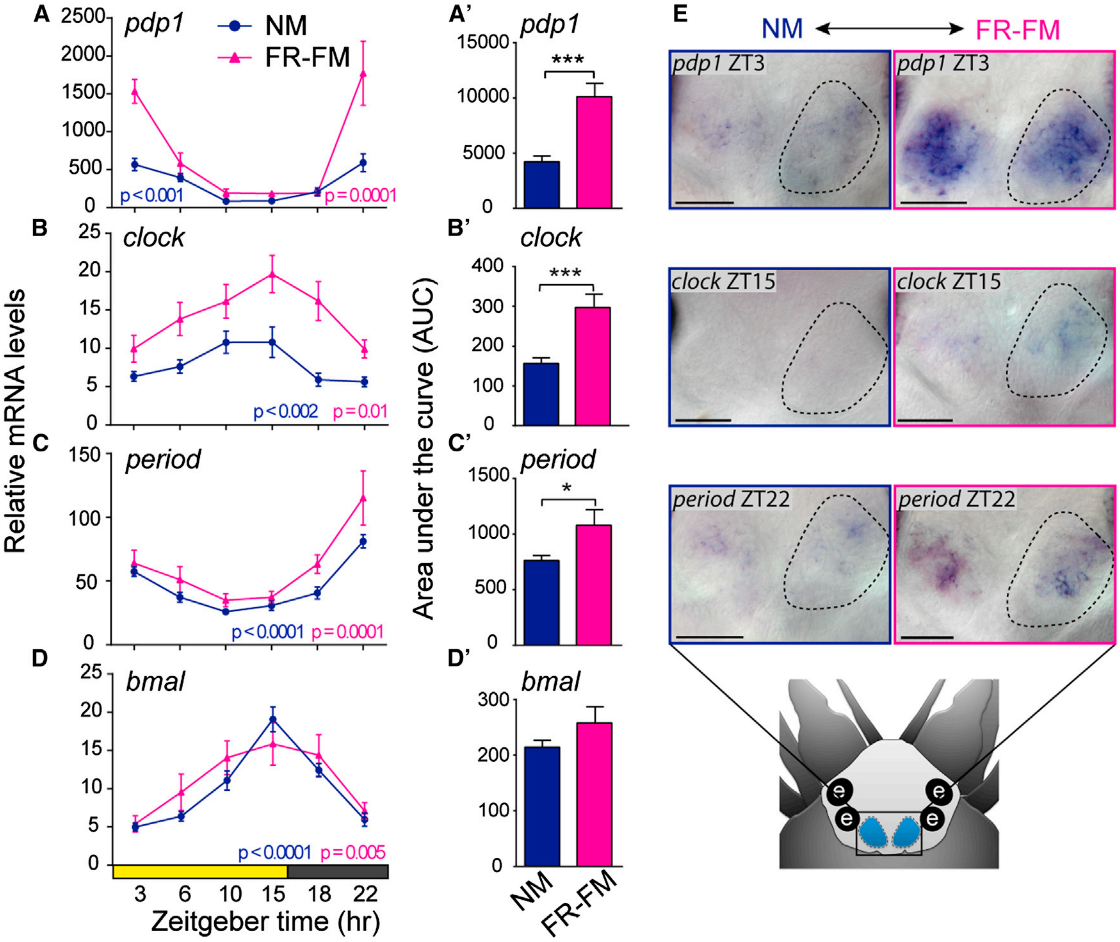

Figure 5. The Circalunar Clock Influences Circadian Clock Gene Expression

(A–D) Temporal profiles of clock gene RNA expression in *Platynereis* heads sampled during NM (blue) and FR-FM (pink) at the indicated Zeitgeber time point (ZT) are shown. See Figure 1C for detailed information on the circalunar-light regimen. Values are means ± SEM, NM n = 5–16, FR-FM n = 3–10; four to five heads per n. The p value was determined by one-way ANOVA.

(A′–D′) Overall daily transcript levels calculated as area under the curve (AUC) based on 24 hr expression data shown in (A)–(D) are shown. Values are means ± SEM; NM n = 6–16, FR-FM n = 3–10. The p value was determined by one-way ANOVA. Significant differences were determined by Wilcoxon signed rank test (*p < 0.05; ***p < 0.001); four to five heads per n. (E) Whole mount in situ hybridization shows an increase of *pdp1*, *clock*, and *period* levels at FR-FM versus NM in the oval shaped circadian clock gene expressing forebrain domain (compare Figures 3A–3E). See Figure S5 for analyses of additional circadian clock genes.

number of worms exhibiting behavioral rhythms with periods clearly different from 24 hr was strongly increased in FR-FM (LD and DD) compared with NM (LD and DD) (Figures 4C and 4L–4N). This provides strong evidence that the circalunar clock affects circadian behavior.

The Circalunar Clock Impacts Transcript Levels of *clock*, *period*, *pdp1*, and *timeless*

Changes in circadian behavior have been directly connected to changes in circadian clock gene levels in *Drosophila* and mice (Antoch et al., 1997; Benito et al., 2007; Kadener et al., 2008). We therefore next investigated if the oscillation and levels of circadian clock gene orthologs were also affected by the circalunar clock by comparing RNA levels between NM and FR-FM (cf. Figure 1C). For the genes *pdp1*, *clock*, *period*, and *timeless*, the circadian expression dynamics (period lengths and phase, represented as shape of the graphs) under FR-FM were not detectably different to NM conditions (Figures 5A-5C; Figure S5A, pink graphs). However, their overall transcript levels were significantly elevated at FR-FM compared to NM (Figures 5A–5C and 5A′–5C′; Figures S5A and S5A′). In contrast, expression levels and circadian dynamics of *bmal* (Figures 5D and 5D′), *tr-cry*, *vrille*, and *timeout* (Figures S5B–S5D and S5B′–S5D′) did not differ between FR-FM and NM conditions (pink versus blue graphs).

We hence conclude that the overall transcript levels of *clock*, *period*, *pdp1*, and *timeless* are directly or indirectly modulated by the circalunar clock.

If this is indeed the case, the transcript levels at the next NM under circalunar free-running conditions (FR-NM; see Figure 1C) should return to the levels observed under normal NM. This is indeed the case for the three genes tested representatively. Circadian oscillations and transcript levels of *clock*, *period*,

and *bmal* in FR-NM resembled that of NM (Figures S5F–S5H and S5F′–S5H′).

Analyses of premature adult brains using WMISH revealed that the elevation of *clock*, *period*, *pdp1*, and *timeless* transcripts during FR-FM was not due to additional brain domains expressing these genes, but that the same cells in the two core circadian brain nuclei now express at higher levels (Figure 5E; Figure S5E).

These results predict that at least one of the circadian clock genes *clock*, *period*, *pdp1*, or *timeless* function either downstream of the circalunar oscillator, or as part of it, and establish the regulation of mRNA levels as an output of the circalunar clock.

Circadian Clock Gene Oscillations Are Not Required for Circalunar Clock Function

We next asked if the circadian clock affects, or is part of, the worm's circalunar clock. Different hypotheses have been put forward to explain rhythms with a semilunar or lunar period length. Many of these models involve circadian oscillators. One model relies on the interaction of the circadian oscillator with an oscillator running with a circalunidian or tidal period (i.e., 24.8 hr or 12.4 hr) so that both only coincide once per lunar or semilunar month (Figure 6A; Soong and Chang, 2012). Alternatively, the counting of circadian cycles has been proposed in the frequency demultiplication hypothesis to lead to a circalunar rhythmic output (Soong and Chang, 2012).

We thus next tested if circadian clock gene oscillations were required for circalunar clock function in *Platynereis*. For this, we interfered with the *Platynereis* circadian clock and assessed the effects of this interference on circalunar spawning peaks. Mammalian casein kinase 1δ/ε and its *Drosophila* ortholog Double time (DBT) are crucial for normal circadian clock function (Lee et al., 2009). Their best-documented function is Period phosphorylation, which serves to enhance Period degradation in both systems (Gallego and Virshup, 2007). PF-670462 and other CK1δ/ε inhibitors severely affect the circadian period in mammalian cells (Eide et al., 2005; Walton et al., 2009).

The *Platynereis ck1δ/ε* ortholog is widely expressed, including in areas of the medial forebrain and the oval- shaped core circadian clock brain nuclei (Figure S3L). Upon PF-670462 treatment, the amplitudes of *bmal*, *clock*, *tr-cry*, *timeout*, *timeless*, and *pdp1* transcriptional cycling were flattened to a level that no clear oscillations were observable anymore in *Platynereis* (Figures 6B and 6C; Figures S6A–S6D), while *period* transcription showed irregular fluctuations (Figure S6E).

Consistent with the abolished molecular circadian clock oscillations, we also found that 70% of PF-670462-treated worms were arrhythmic in their daily activity when tested under NM(LD) conditions (Figures 6D–6G). The remaining 30% showed weak rhythmicity, but their period length was severely altered to about 17 hr. Despite their severely disrupted circadian rhythmicity, PF-670462-treated worms were still capable of displaying all types of normal behaviors (Figure 6D). This is apparent from the mean analysis (Figure 6E), but also from individual worms (Figures 6D–6G), attesting to the notion that PF-670462 treatment leads to a disruption of the circadian core clock in *Platynereis* in the majority of the population. Despite these significant changes in circadian clock gene dynamics, however, PF-670462 treatment did not affect the circalunar spawning periodicity of *Platynereis* when compared to controls in free-running experiments (Figures 6H and 6I; compare to Figure 1H for arrhythmic spawning).

We tested several concentrations of PF-670462 and performed the circalunar spawning assays with the lowest concentration still exhibiting robust effects on circadian clock molecular oscillations. Whereas we cannot exclude that PF-670462 also affects other targets at the given concentration, we can conclude that none of these effects, including the one on the circadian clock, shows an obvious impact on the circalunar clock.

Based on these results, we conclude that the circalunar clock in *Platynereis* is independent of the oscillations of the circadian transcriptional clock (Figure 7).

DISCUSSION

Life with More Than One Type of Clock

Here, we show that the bristle worm *Platynereis dumerilii* harbors two endogenous clocks, with a circadian and a circalunar period length, respectively. The coexistence of multiple clocks in one organism is likely a rather common phenomenon, yet most clearly displayed outside of the group of the conventional molecular animal model species (Naylor, 2010; Tessmar-Raible et al., 2011). Consequently, the interactions of such clocks have only been investigated to a very limited extent (Takekata et al., 2012). We provide evidence that the oscillatory mechanisms of both clocks are distinct, but that they both converge on the regulation of transcript levels and behavior.

Whereas our behavioral analyses focused on premature adult *Platynereis* worms, we propose that the observed modulation of circadian behavior by the circalunar clock also underlies the regulation of other behaviors, such as the nuptial dance of mature animals. This mating behavior is known to be synchronized both to particular days of the month and to specific hours of the night (Korringa, 1947). Synchronized mating likely increases the reproductive success of externally fertilizing animals, especially when they occur in large populations, as for instance in reef corals (Harrison et al., 1984).

The biological implication of the changes in behavioral period length of the premature adult worms might only be understandable when we will know more about the natural conditions the worms have to adapt to outside of the time of mating.

Parallel to our study, work on *Eurydice pulchra* revealed the coexistence of molecularly independent circatidal and circadian clocks in this crustacean (Zhang et al., 2013). A possible coordination of these two clocks might occur by their coregulation by CK1δ/ε, as PF-670462 incubation led to an increased period length of both circadian and circatidal clocks (Zhang et al., 2013). An effect of PF-670462 on the period length of the *Platynereis* circalunar clock is possible, but as *Platynereis* only spawns once, our current analyses rely on scoring large populations, making this question technically very difficult to test. Live readouts of the circalunar clock in individual worms will be helpful to answer such and further questions on circalunar and circadian clock interactions in the future.

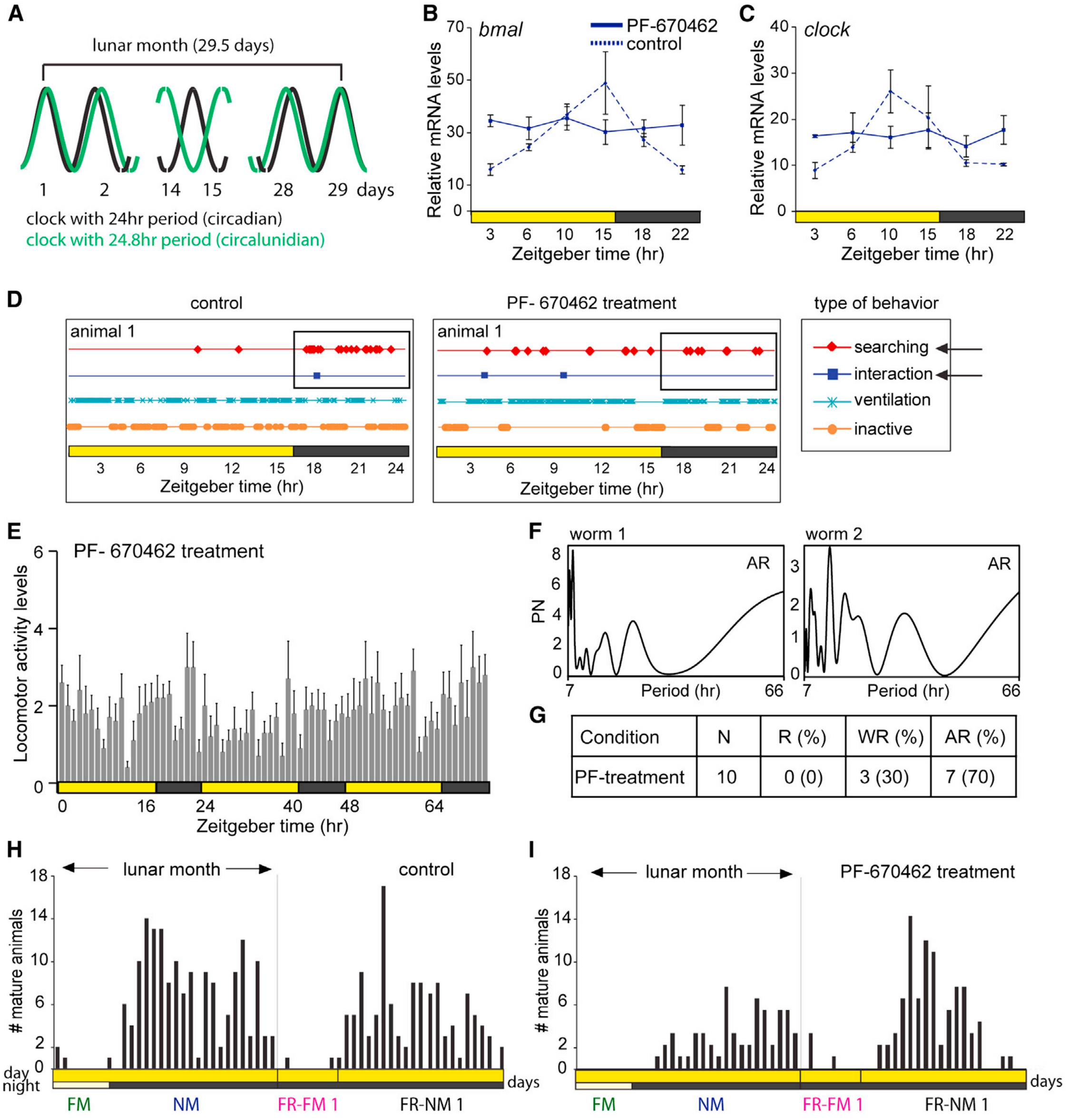

Figure 6. The Circalunar Clock Is Independent of Circadian Clock Oscillations

(A) A dual oscillator model could explain circalunar clock function. A circadian (24 hr, length of the solar day) and circalunidian (24.8 hr, length of a lunar day) oscillator function together to generate monthly (29.5 days) periods.

(B and C) Circadian clock gene transcriptional oscillations are severely affected under PF-670462 treatment compared to nontreated controls (dashed line). Values are means ± SEM; n = 3; four to five heads per n. See Figure S6 for additional circadian clock genes.

(D) Behavioral analyses (one behavioral score per minute of a 10 min interval per hour) as described in Figures S4A and S4B from one representative example of untreated controls (active behavior, indicated by arrows, mainly restricted to the dark phase) versus PF-670462-treated worms (active behavior distributed).

(E) PF-670462 abolishes rhythmic circadian locomotor activity in *Platynereis*. Worms were recorded under 16:8LD circadian illumination (see Figure 4A for a nontreated comparison).

(F and G) Periodogram analyses of individual worms show that PF-670462 treated animals are in majority arrhythmic (AR). No worm was rhythmic (R), and few worms remaining weakly rhythmic (WR) showed a strongly altered period length of 17 hr.

(H and I) Circalunar spawning cycles are maintained in control (H) and under PF-670462 treatment (I). Collection data from five independent experiments were pooled.

The Effect of the Circalunar Clock on Circadian Period Length

Our results show that on the behavioral level, the period length and strength of the circadian rhythm are significantly modulated by the circalunar clock. The change in behavioral period length contrasts with the seemingly unaltered period length of the molecular oscillations of the core circadian clock genes. We currently see two possibilities to explain this discrepancy.

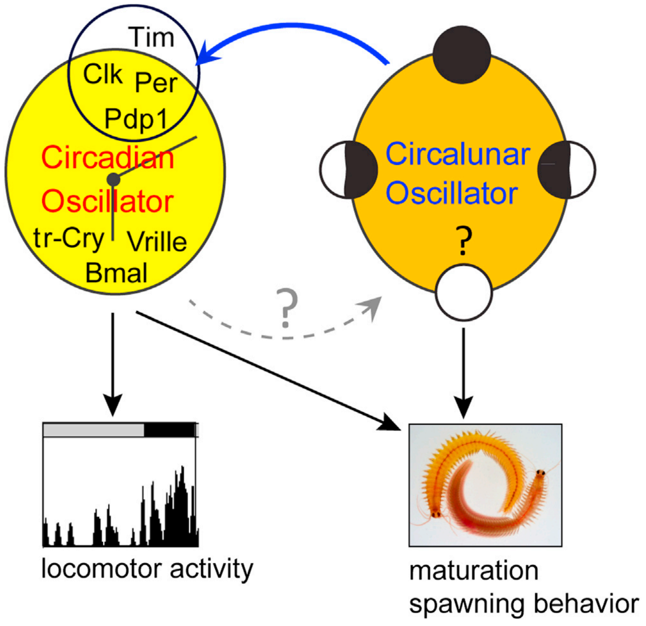

Figure 7. Circadian and Circalunar Clock Model in *Platynereis*

Proposed interaction of separate circadian and circalunar oscillators in *Platynereis dumerilii* is shown. Solid blue line indicates impact of the circalunar oscillator on the transcriptional regulation of circadian clock gene expression resulting in elevated levels of *pdp1*, *period*, *clock*, and *timeless*. The impact of the circalunar clock on the circadian clock genes can be direct or indirect on one or all of these genes.

On the one hand, the period length of the worm's locomotor rhythms could be modulated independently of the core circadian clock, albeit still also under circadian clock control. In such a model, the circalunar clock would directly target genes (downstream or independently of the circadian clock) that can regulate behavior. It is for instance conceivable that the circalunar clock affects the levels of hormone precursors, processing enzymes or neurotransmitters. By changing thresholds, these changes (in combination with the circadian clock control) could subsequently result in the observed behavioral phenotype. To exemplify, if lowering the overall levels of a suppressor, a transmitter affecting behavioral activity could reach critical levels high enough to elicit activity more often (e.g., twice per day instead of once per day).

On the other hand, it could be possible that the elevation of *clock*, *period*, *pdp1*, and *timeless* mRNA levels during FR-FM causes (at least partly) the behavioral changes. A possible scenario how this could be the case is outlined below.

It is well-established that 12 hr rhythms occur in the expression of approximately 1% of all genes in mouse liver, although the circadian clock is unaltered (Hughes et al., 2009, 2012; Vollmers et al., 2009). In addition, 8 hr rhythms in gene expression also occur naturally (Hughes et al., 2009). It seems plausible that what happens in the liver might also happen to cells in other tissues, such as neurons in the brain. In addition, it is also plausible that changes in locomotor activity rhythms can be controlled by changes in gene activity of genes affecting behavior, such as hormonal precursors, processing enzymes, or neurotransmitters. Thus, gene activity cycling with 12 hr or 8 hr rhythms could generate 12 hr or 8 hr behavioral activity cycles in the background of a normally functioning circadian clock.

A recent theoretical work provides a mathematical model explaining the generation of such naturally occurring 12 hr gene expression cycles based on changes in the binding of circadian transcription factors to separate (noncompetitive) binding sites (Westermark and Herzel, 2013). More specifically, two points of the mathematical model might help to explain the findings described in our work. (1) The oscillation amplitudes of the core circadian transcription factors have an impact on the circadian term of the equation (i.e., if they are equal, the circadian term will vanish). In other words, 12 hr cycles can occur based on changes in the amplitude of the core circadian transcription factors that themselves still cycle with a 24 hr periodicity. This could explain, how the changes in transcript levels we observe for some core circadian transcription factors could finally lead to changes in locomotor activity cycles. (2) A combination of less 24 hr and more 12 hr periods in transcription factor rhythms can produce 8 hr fluctuations. In both FR-FM (DD and LD) conditions, we observe such a decrease of 24 hr behavioral periods, combined with an increase in 12 hr periods. Thus, our observed combination might "automatically" lead to the appearance of ~8 hr rhythms, which is what we indeed observed.

Furthermore, besides the mathematical-model-based considerations, there is also functional evidence that slight changes in gene levels can influence the period length of locomotor activity. The introduction of one or more additional copies of the *clk* genomic region significantly alters the circadian locomotor activity period in *Drosophila* (Kadener et al., 2008). This effect is thought to be caused by the increased transcriptional levels of *clk*'s direct target genes *per*, *pdp1*, and *tim* (Kadener et al., 2008). Remarkably, we see the same genes upregulated by the circalunar clock in *Platynereis*, raising the possibility that, in analogy to *Drosophila*, an increase in RNA levels of *Platynereis clock* can account for the upregulation of *period*, *pdp1*, and *timeless* transcript levels, and in consequence for the significant shortening of the circadian behavioral period length of the worm. One additional piece of evidence that changes in circadian clock gene mRNA levels can manifest themselves in changes in locomotor output rhythms stems from a study of the *pdp1* gene (Benito et al., 2007).

Finally, it should also be taken into consideration that the changes in locomotor period length are differently prominent in different individual animals. While we can observe individual difference on behavioral levels, the observation of gene activity in individual animal heads (and not in pools of animal heads) over time is currently technically not feasible. This could blur smaller changes in the period length of the molecular oscillations.

Possible Circalunar Clock Models in *Platynereis dumerilii*

Our study shows that circalunar clock function is not affected even when the transcriptional oscillations of putative circadian clock genes are severely impaired, arguing against any circalunar clock model involving the classical circadian clock. It is,

however, conceivable that the maintained daily light-dark cycle is sufficient to drive circalunar rhythms, in absence of circadian clock oscillations. Finally, our data do not test if the classical circadian clock might still be required for the entrainment of the circalunar oscillator.

EXPERIMENTAL PROCEDURES

Worm Culture and Light Conditions

Worms were maintained as described previously (Hauenschild and Fischer, 1969). See the Extended Experimental Procedures for further detail. Worms of the following inbred strains were used: PIN-mix, VIO-mix, and ORA-mix. All animal work was conducted according to Austrian and European guidelines for animal research.

Gene Identification

Fragments of *Platynereis* sequences described in this study were identified by high-throughput sequencing of normalized complementary DNA (cDNA) using 454 technology. These fragments were subsequently expanded by rapid amplification of cDNA end (RACE) PCR, using Clontech's Smart RACE cDNA amplification kit. Primers and program are listed in the Extended Experimental Procedures.

Phylogenetic Analyses

Sequences were aligned using the MAFFT alignment algorithm. (http://www.ebi.ac.uk/Tools/mafft/index.html). The resulting alignments were subsequently used to generate NJ and ML trees. See the Extended Experimental Procedures for further detail.

Total RNA Extraction and RT-PCR

Total RNA was extracted from heads of premature adult worms using the RNeasy Mini Kit (QIAGEN). Reverse transcription was carried out using 0.4 μg of total RNA as template (QuantiTect Reverse Transcription kit, QIAGEN). RT-PCR analyses were performed using a Step-One-Plus cycler. The expression of each test gene was normalized by the amount of the internal control gene *cdc5*. Using *rps9* as reference genes made no significant difference. The relative expression was calculated using the following formula: $1/2^{\Delta Ct}$. Overall levels of expression (area under the curve) were calculated using the trapezoid rule on the relative expression profile of any given gene over 24 hr. All data are shown as the mean ± SEM. See Extended Experimental Procedures for primers and program.

Behavioral Observations and Analyses

Animals entrained under circadian and circalunar light regimes for at least 2 month were transferred into a box (20 × 20 cm, 15–20 animals) containing saltwater (depth 1 cm). Animals were fed prior to the recording to eliminate any behavioral changes in response to feeding. Locomotor behavior was recorded within a black box (white light light-emitting diodes [LEDs]: COINlight CM01E, 150 lux; see spectral analysis in Figure S4C) under given light regimen (LD, DD) using a Chameleon USB 2.0 digital video camera. Light intensity was measured with a USB2000+ spectrometer (Ocean Optics). In order to visualize the worms under dark conditions, an infrared-light LED array (Roschwege GmbH) (990 nm) was placed inside the black box and an infrared (IR) high-pass filter restricted to the detection of IR light into to the camera. Video images were taken continuously over several days and used to evaluate the behavior according to the specified types of behavior (active = 1, inactive = 0).

Behavior was analyzed manually every 1 min of a 10 min interval per hour and the data were imported into ActogramJ Software (University of Wuerzburg) for circadian analysis (Schmid et al., 2011). Locomotor activity was calculated as the number of active behavior events occurring every 1 hr. Periodograms were generated using Lomb-Scargle analysis. Periodicities were confirmed using Fourier transform analysis (FFT) and chi-square analysis. The significant p level was set to 0.05. Worms with a power $\geq$ 15 were designated as rhythmic (R), worms with a power $\leq$ 15 were designated as weakly rhythmic (WR). Worms with a power $\leq$ p level in periodogram analysis were defined as arrhythmic (AR). For time-point analysis, a t test was performed using GraphPad Prism version 6.00 for Windows.

Light-Induced Degradation Assay

Full-length *Pdu-tr-cry* and *Pdu-L-cry* sequences were codon optimized for insect codon usage and subcloned at the NotI/XbaI restriction sites into pAC5.1/V5-HisA by Entelechon. As the positive/negative controls, monarch butterfly *Danaus plexippus Dp-Cry1* and *2* were used (Zhu et al., 2005). *Dp-Cry1* and *2* subcloned into pAC5.1/V5-HisA were kindly supplied by Dr. Reppert. pAct-EGFP, in which *Drosophila actin* promoter, EGFP, and SV40 polyA sequences were subcloned into pBlueScript (Invitrogen), was used for internal control of transfection. S2 cells were maintained at 25°C in Schneider's *Drosophila* medium (Invitrogen) supplemented with 10% heat-inactivated fetal bovine serum (Biological Industries). S2 cells (1.5 × 10^6) were seeded in six-well plates and next-day transfection was performed using Cellfectin reagent (Invitrogen). Each transfection had 4 μg of each *Pdu-tr-cry*, *Pdu-L-cry*, *Dp-Cry1*, or *Dp-Cry2*, and 1 μg of *pAct-EGFP* was added. Then 48 hr after transfection, light treatment was performed as described previously (Yuan et al., 2007). Light treatment involved placing S2 cell culture plate under fluorescent light (3,000–4,500 lux) for 6 hr at 24°C. Dark control plate was wrapped with aluminum foil and incubated beside the light-treated plate. Western blotting was performed using a monoclonal mouse anti-V5 immunoglobulin G (IgG) (Nacalai Tesque) and a monoclonal mouse anti-GFP IgG (Roche Diagnostics). Bands intensity was measured by LAS1000 (FUJIFILM). The cryptochrome's (V5) band intensity was normalized by each GFP band's intensity.

Transcription Repression Assay

Full-length *Pdu-clock, Pdu-bmal* and *Pdu-6-4photolyase* sequences were PCR amplified from cDNA, subcloned into pJet2.1, sequence verified and subsequently subcloned into pAC5.1/V5-HisA, generating pAct-*Pdu-clock*, pAct-*Pdu-bmal* and pAct-*Pdu-6-4photolyase*.

To generate the reporter construct, a 120 bp segment of the 5′ flanking region of monarch butterfly, *Danaus plexippus*, *per2* gene (NCBI accession number AY364479, bases 1,177–1,296), which contains two E-boxes, was synthesized and cloned in the pGL3-Basic vector (Promega), generating plasmid p*DpPer2* (E-box)-luc. S2 cells (6 × 10^5) were seeded in 12-well plates and transfected the next day with Cellfectin (Invitrogen). Each transfection had 350 ng each of pAct-*Pdu-clock*, pAct-*Pdu-bmal*, and various amounts of pAct-*Pdu-tr-cry* or pAct-*Pdu-L-cry* or pAct-*Pdu-6-4photolyase* or 350 ng of pAct-*Dpcry1* or *2* (Kume et al., 1999). In each transfection experiment, the reporter plasmid p*DpPer2*-luc (10 ng) and the p*RL*-SV40 vector (Promega) (25 ng) were added (Kobayashi et al., 2000). The total DNA per well was adjusted to1.05 μg by adding pAC5.1/V5-HisA as carrier. Then 48 hr after transfection, cells were harvested and their firefly and *Renilla* luciferase activities determined by luminometry. The reporter luciferase activity was normalized for each sample by determining the firefly:*Renilla*luciferase activity ratios. In each experiment, the luciferase activity of the PduClk:PduBMAL1-containing sample was taken as 100%. All experiments were repeated at least three times.

Statistical Analysis

Statistical analysis of real-time data was performed using the nonparametric Wilcoxon signed rank test using *R Software: A Language and Environment for Statistical Computing*, providing a conservative test for significant differences between two sample types (http://www.R-project.org) (Hollander and Wolfe, 1973).

For the Wilcoxon signed rank test, a paired, one-tailed significance interval of 0.05 was used (*p < 0.05, **p < 0.005, ***p < 0.001, ****p < 0.0001).

One-way ANOVA test and Student's t test was performed using GraphPad Prism version 6.00 for Windows (R Development Core Team, 2005).

Treatment of Worms with PF-670462

Premature adult worms of mixed ages were incubated in 800 μM PF-670462 (Tocris, #3316) and grown as the rest of the worm culture. Water was changed and new drug added every week. After 2 weeks of continuous treatment, worms were incubated repeatedly for 5 days in 800 μM PF-670462 and in fresh seawater for 2 days to avoid possible side effects. Treatment was always continuous during the FR-FM phase. PF-670462 is dissolved in water. Thus,

control animals were cultured under the same conditions (same room, light cycle, moon cycle, water change, feeding), but not incubated in PF-670462.

Whole-Mount In Situ Hybridization

Platynereis WMISH was performed according to Tessmar-Raible et al. (2005), with the modifications for adult heads outlined in Backfisch et al. (2013).

Immunocytochemistry

Monoclonal anti-mouse anti-acetylated α-tubulin (clone no. 6-11B-1; Sigma-Aldrich. T6793) was used in a 1:200 dilution as previously described (Arendt et al., 2004).

Mounting and Microscopy

Platynereis adult heads were mounted in 90% glycerol. See Extended Experimental Procedures for details.

ACCESSION NUMBERS

Gene and genomic sequences have been deposited into the NCBI Genbank under the accession numbers GU322428, GU322429, GU322430, GU322431, KF316921, KF316922, KF316923, KF316924, KF316925, KF316926, KF316927, KF316928, KF316929, KF316930, and KF316931.

SUPPLEMENTAL INFORMATION

Supplemental Information includes Extended Experimental Procedures and six figures and can be found with this article online at http://dx.doi.org/10.1016/j.celrep.2013.08.031.

ACKNOWLEDGMENTS

The authors wish to thank Barbara Helm, Rodolfo Costa, and four anonymous reviewers for constructive feedback on the manuscript as well as Steven Reppert and Michael Rosbash for constructive feedback on its initial stage. Barbara Helm also gave very valuable input for the interpretation of the locomotor activity data analyses. The authors thank Florian Raible, Stephanie Bannister, Ruth Fischer, and Sven Schenk for discussions on the work and manuscript and Gerald Nyakatura and Berthold Fartmann (LGC Genomics) for expert technical assistance. Steven Reppert kindly provided the plasmids encoding V5-tagged *dp*Cry1 and *dp*Cry2. This work was supported by funds from the Max F. Perutz Laboratories and the University of Vienna, the research platform "Marine Rhythms of Life" of the University of Vienna, an FWF START award (#AY0041321; to K.T-R.), funds from the IMP (to A.D.S.), and an HFSP research grant (#RGY0082/2010; to K.T.-R. and T.I.-F.).

Received: February 6, 2013
Revised: July 3, 2013
Accepted: August 28, 2013
Published: September 26, 2013

REFERENCES

Antoch, M.P., Song, E.J., Chang, A.M., Vitaterna, M.H., Zhao, Y., Wilsbacher, L.D., Sangoram, A.M., King, D.P., Pinto, L.H., and Takahashi, J.S. (1997). Functional identification of the mouse circadian Clock gene by transgenic BAC rescue. Cell *89*, 655–667.

Arendt, D., Tessmar-Raible, K., Snyman, H., Dorresteijn, A.W., and Wittbrodt, J. (2004). Ciliary photoreceptors with a vertebrate-type opsin in an invertebrate brain. Science *306*, 869–871.

Aschoff, J.E. (1981). In Handbook of Behavioral Neurobiology, *Volume 4* (New York: Plenum Press).

Bachleitner, W., Kempinger, L., Wülbeck, C., Rieger, D., and Helfrich-Förster, C. (2007). Moonlight shifts the endogenous clock of *Drosophila melanogaster*. Proc. Natl. Acad. Sci. USA *104*, 3538–3543.

Backfisch, B., Veedin Rajan, V.B., Fischer, R.M., Lohs, C., Arboleda, E., Tessmar-Raible, K., and Raible, F. (2013). Stable transgenesis in the marine annelid *Platynereis dumerilii* sheds new light on photoreceptor evolution. Proc. Natl. Acad. Sci. USA *110*, 193–198.

Benito, J., Zheng, H., and Hardin, P.E. (2007). PDP1epsilon functions downstream of the circadian oscillator to mediate behavioral rhythms. J. Neurosci. *27*, 2539–2547.

Benna, C., Bonaccorsi, S., Wülbeck, C., Helfrich-Förster, C., Gatti, M., Kyriacou, C.P., Costa, R., and Sandrelli, F. (2010). *Drosophila timeless2* is required for chromosome stability and circadian photoreception. Curr. Biol. *20*, 346–352.

Blau, J., and Young, M.W. (1999). Cycling vrille expression is required for a functional Drosophila clock. Cell *99*, 661–671.

Chaves, I., Pokorny, R., Byrdin, M., Hoang, N., Ritz, T., Brettel, K., Essen, L.O., van der Horst, G.T., Batschauer, A., and Ahmad, M. (2011). The cryptochromes: blue light photoreceptors in plants and animals. Annu. Rev. Plant Biol. *62*, 335–364.

Ciarleglio, C.M., Axley, J.C., Strauss, B.R., Gamble, K.L., and McMahon, D.G. (2011). Perinatal photoperiod imprints the circadian clock. Nat. Neurosci. *14*, 25–27.

Cold Spring Harbor Symposia on Quantitative Biology. (2007). In Clocks and Rhythms, *Volume 72* (Cold Spring Harbor: Cold Spring Harbor Laboratory Press).

Cyran, S.A., Buchsbaum, A.M., Reddy, K.L., Lin, M.C., Glossop, N.R., Hardin, P.E., Young, M.W., Storti, R.V., and Blau, J. (2003). vrille, Pdp1, and dClock form a second feedback loop in the Drosophila circadian clock. Cell *112*, 329–341.

Darlington, T.K., Wager-Smith, K., Ceriani, M.F., Staknis, D., Gekakis, N., Steeves, T.D., Weitz, C.J., Takahashi, J.S., and Kay, S.A. (1998). Closing the circadian loop: CLOCK-induced transcription of its own inhibitors per and tim. Science *280*, 1599–1603.

Dupré, S.M., and Loudon, A.S. (2007). Circannual clocks: annual timers unraveled in sheep. Curr. Biol. *17*, R216–R217.

Eide, E.J., Woolf, M.F., Kang, H., Woolf, P., Hurst, W., Camacho, F., Vielhaber, E.L., Giovanni, A., and Virshup, D.M. (2005). Control of mammalian circadian rhythm by CKIepsilon-regulated proteasome-mediated PER2 degradation. Mol. Cell. Biol. *25*, 2795–2807.

Emery, P., Stanewsky, R., Helfrich-Förster, C., Emery-Le, M., Hall, J.C., and Rosbash, M. (2000). *Drosophila* CRY is a deep brain circadian photoreceptor. Neuron *26*, 493–504.

Fage, L., and Legendre, R. (1927). Peches planctoniques à la lumière effectuées à Banyuls-sur-mer et à Concarneau, I, Annelides Polychetes. Arch. Zool. Exp. Gén. Paris *67*, 23–222.

Fischer, A. (1969). [A pigment deficient mutant in the polychaete Platynereis dumerilii]. Mol. Gen. Genet. *104*, 360–370.

Fox, H.M. (1924). Lunar periodicity in reproduction. Proc. R. Soc. Lond. B *95*, 523–550.

Franke, H.D. (1985). On a clocklike mechanism timing lunar-rhythmic reproduction inTyposyllis prolifera (Polychaeta). J. Comp. Physiol. A Neuroethol. Sens. Neural Behav. Physiol. *156*, 553–561.

Fukushiro, M., Takeuchi, T., Takeuchi, Y., Hur, S.P., Sugama, N., Takemura, A., Kubo, Y., Okano, K., and Okano, T. (2011). Lunar phase-dependent expression of cryptochrome and a photoperiodic mechanism for lunar phase-recognition in a reef fish, goldlined spinefoot. PLoS ONE *6*, e28643.

Gallego, M., and Virshup, D.M. (2007). Post-translational modifications regulate the ticking of the circadian clock. Nat. Rev. Mol. Cell Biol. *8*, 139–148.

Gekakis, N., Staknis, D., Nguyen, H.B., Davis, F.C., Wilsbacher, L.D., King, D.P., Takahashi, J.S., and Weitz, C.J. (1998). Role of the CLOCK protein in the mammalian circadian mechanism. Science *280*, 1564–1569.

Glossop, N.R., Houl, J.H., Zheng, H., Ng, F.S., Dudek, S.M., and Hardin, P.E. (2003). VRILLE feeds back to control circadian transcription of Clock in the Drosophila circadian oscillator. Neuron *37*, 249–261.

Gotter, A.L. (2006). A Timeless debate: resolving TIM's noncircadian roles with possible clock function. Neuroreport *17*, 1229–1233.

Harrison, P.L., Babcock, R.C., Bull, G.D., Oliver, J.K., Wallace, C.C., and Willis, B.L. (1984). Mass spawning in tropical reef corals. Science *223*, 1186–1189.

Hauenschild, C. (1954). Ueber die lunarperiodische Schwärmen von *Platynereis dumerilii* in Laboratorienzuchten. Naturwissenschaften *41*, 556–557.

Hauenschild, C. (1955). Photoperiodizität als ursache des von der mondphase abhangigen metamorphose-rhythmus bei dem polychaeten *Platynereis dumerilii*. Z. Naturforsch. B *10*, 658–662.

Hauenschild, C. (1960). Lunar periodicity. Cold Spring Harb. Symp. Quant. Biol. *25*, 491–497.

Hauenschild, C. (1964). Postembryonale Entwicklungssteuerung durch ein Gehirn-Hormon bei *Platynereis dumerilii*. Zool. Anz. *27*, 111–120.

Hauenschild, C. (1966). Der hormonale Einfluß des Gehirns auf die sexuelle Entwicklung bei dem Polychaeten *Platynereis dumerilii*. Gen. Comp. Endocrinol. *6*, 26–73.

Hauenschild, C., and Fischer, A. (1969). *Platynereis dumerilii*. Mikroskopische Anatomie, Fortpflanzung, Entwicklung. Großes Zoologisches Praktikum *10b*, 1–54.

Hazlerigg, D.G., and Lincoln, G.A. (2011). Hypothesis: cyclical histogenesis is the basis of circannual timing. J. Biol. Rhythms *26*, 471–485.

Hofmann, D.K. (1975). Analyse der beziehungen zwischen regenerationsleistung, geschlechtsreifung und endokrinem system bei *Platynereis dumerilii* (Annelida: Polychaeta). Verh. Dtsch. Zool. Ges., 314–319.

Hollander, M., and Wolfe, D.A. (1973). Nonparametric Statistical Methods (New York: John Wiley and Sons).

Hughes, M.E., DiTacchio, L., Hayes, K.R., Vollmers, C., Pulivarthy, S., Baggs, J.E., Panda, S., and Hogenesch, J.B. (2009). Harmonics of circadian gene transcription in mammals. PLoS Genet. *5*, e1000442.

Hughes, M.E., Hong, H.K., Chong, J.L., Indacochea, A.A., Lee, S.S., Han, M., Takahashi, J.S., and Hogenesch, J.B. (2012). Brain-specific rescue of Clock reveals system-driven transcriptional rhythms in peripheral tissue. PLoS Genet. *8*, e1002835.

Hunter-Ensor, M., Ousley, A., and Sehgal, A. (1996). Regulation of the Drosophila protein timeless suggests a mechanism for resetting the circadian clock by light. Cell *84*, 677–685.

Ivanchenko, M., Stanewsky, R., and Giebultowicz, J.M. (2001). Circadian photoreception in Drosophila: functions of cryptochrome in peripheral and central clocks. J. Biol. Rhythms *16*, 205–215.

Kadener, S., Menet, J.S., Schoer, R., and Rosbash, M. (2008). Circadian transcription contributes to core period determination in Drosophila. PLoS Biol. *6*, e119.

Kaiser, T.S., Neumann, D., and Heckel, D.G. (2011). Timing the tides: genetic control of diurnal and lunar emergence times is correlated in the marine midge *Clunio marinus*. BMC Genet. *12*, 49.

Kobayashi, Y., Ishikawa, T., Hirayama, J., Daiyasu, H., Kanai, S., Toh, H., Fukuda, I., Tsujimura, T., Terada, N., Kamei, Y., et al. (2000). Molecular analysis of zebrafish photolyase/cryptochrome family: two types of cryptochromes present in zebrafish. Genes Cells *5*, 725–738.

Korringa, P. (1947). Relations between the moon and periodicity in the breeding of marine animals. Ecol. Monogr. *17*, 347–381.

Koštál, V. (2011). Insect photoperiodic calendar and circadian clock: independence, cooperation, or unity? J. Insect Physiol. *57*, 538–556.

Krishnan, B., Levine, J.D., Lynch, M.K., Dowse, H.B., Funes, P., Hall, J.C., Hardin, P.E., and Dryer, S.E. (2001). A new role for cryptochrome in a *Drosophila* circadian oscillator. Nature *411*, 313–317.

Kume, K., Zylka, M.J., Sriram, S., Shearman, L.P., Weaver, D.R., Jin, X., Maywood, E.S., Hastings, M.H., and Reppert, S.M. (1999). mCRY1 and mCRY2 are essential components of the negative limb of the circadian clock feedback loop. Cell *98*, 193–205.

Last, K.S. (2003). An actograph and its use in the study of foraging behaviour in the benthic polychaete, *Nereis virens* (Sars). J. Exp. Mar. Biol. Ecol. *287*, 237–248.

Last, K.S., and Olive, P.J. (2004). Interaction between photoperiod and an endogenous seasonal factor in influencing the diel locomotor activity of the benthic polychaete Nereis virens Sars. Biol. Bull. *206*, 103–112.

Lee, H., Chen, R., Lee, Y., Yoo, S., and Lee, C. (2009). Essential roles of CKIdelta and CKIepsilon in the mammalian circadian clock. Proc. Natl. Acad. Sci. USA *106*, 21359–21364.

Levine, J.D., Funes, P., Dowse, H.B., and Hall, J.C. (2002). Advanced analysis of a cryptochrome mutation's effects on the robustness and phase of molecular cycles in isolated peripheral tissues of Drosophila. BMC Neurosci. *3*, 5.

Levy, O., Appelbaum, L., Leggat, W., Gothlif, Y., Hayward, D.C., Miller, D.J., and Hoegh-Guldberg, O. (2007). Light-responsive cryptochromes from a simple multicellular animal, the coral *Acropora millepora*. Science *318*, 467–470.

Lin, F.J., Song, W., Meyer-Bernstein, E., Naidoo, N., and Sehgal, A. (2001). Photic signaling by cryptochrome in the *Drosophila* circadian system. Mol. Cell. Biol. *21*, 7287–7294.

Lincoln, G.A., Clarke, I.J., Hut, R.A., and Hazlerigg, D.G. (2006). Characterizing a mammalian circannual pacemaker. Science *314*, 1941–1944.

Myers, M.P., Wager-Smith, K., Wesley, C.S., Young, M.W., and Sehgal, A. (1995). Positional cloning and sequence analysis of the *Drosophila* clock gene, *timeless*. Science *270*, 805–808.

Naylor, E. (2010). Chronobiology of Marine Organisms (Cambridge: Cambridge University Press).

Padmanabhan, K., Robles, M.S., Westerling, T., and Weitz, C.J. (2012). Feedback regulation of transcriptional termination by the mammalian circadian clock PERIOD complex. Science *337*, 599–602.

Palmer, J.D. (1974). A potpourri of lunar-related rhythms. In Biological Clocks in Marine Organisms: The Control of Physiological and Behavioral Tidal Rhythms, J.D. Palmer, ed. (New York: John Wiley and Sons, Inc.), pp. 105–123.

R Development Core Team (2005). R: A Language and Environment for Statistical Computing. Reference index version 2.12.2 (2011-02-25) (Vienna: R Foundation for Statistical Computing).

Ranzi, S. (1931a). Maturita sessuale degli Anellidi e fasi lunari. Boll. Soc. Ital. Biol. Sper. *6*, 18.

Ranzi, S. (1931b). Ricerche sulla biologia sessuale degli Anellidi. Pubbl. Stn. Zool. Napoli *11*, 271–292.

Retzius, G. (1895). Zur Kenntnis des Gehirnganglions und des sensiblen Nervensystems der Polychaeten. Biol. Untersuch. *7*, 6–11.

Roenneberg, T., and Merrow, M. (2005). Circadian clocks - the fall and rise of physiology. Nat. Rev. Mol. Cell Biol. *6*, 965–971.

Sancar, A. (2008). Structure and function of photolyase and in vivo enzymology: 50th anniversary. J. Biol. Chem. *283*, 32153–32157.

Schmid, B., Helfrich-Förster, C., and Yoshii, T. (2011). A new ImageJ plug-in "ActogramJ" for chronobiological analyses. J. Biol. Rhythms *26*, 464–467.

Soong, K., and Chang, Y.H. (2012). Counting circadian cycles to determine the period of a circasemilunar rhythm in a marine insect. Chronobiol. Int. *29*, 1329–1335.

Sugama, N., Park, J.G., Park, Y.J., Takeuchi, Y., Kim, S.J., and Takemura, A. (2008). Moonlight affects nocturnal *Period2* transcript levels in the pineal gland of the reef fish *Siganus guttatus*. J. Pineal Res. *45*, 133–141.

Takekata, H., Matsuura, Y., Goto, S.G., Satoh, A., and Numata, H. (2012). RNAi of the circadian clock gene period disrupts the circadian rhythm but not the circatidal rhythm in the mangrove cricket. Biol. Lett. *8*, 488–491.

Tessmar-Raible, K., Steinmetz, P.R., Snyman, H., Hassel, M., and Arendt, D. (2005). Fluorescent two-color whole mount in situ hybridization in Platynereis dumerilii (Polychaeta, Annelida), an emerging marine molecular model for evolution and development. Biotechniques *39*, 460, 462, 464.

Tessmar-Raible, K., Raible, F., Christodoulou, F., Guy, K., Rembold, M., Hausen, H., and Arendt, D. (2007). Conserved sensory-neurosecretory cell types in annelid and fish forebrain: insights into hypothalamus evolution. Cell *129*, 1389–1400.

Tessmar-Raible, K., Raible, F., and Arboleda, E. (2011). Another place, another timer: Marine species and the rhythms of life. Bioessays *33*, 165–172.

Tomer, R., Denes, A.S., Tessmar-Raible, K., and Arendt, D. (2010). Profiling by image registration reveals common origin of annelid mushroom bodies and vertebrate pallium. Cell *142*, 800–809.

Vanin, S., Bhutani, S., Montelli, S., Menegazzi, P., Green, E.W., Pegoraro, M., Sandrelli, F., Costa, R., and Kyriacou, C.P. (2012). Unexpected features of *Drosophila* circadian behavioural rhythms under natural conditions. Nature *484*, 371–375.

Vollmers, C., Gill, S., DiTacchio, L., Pulivarthy, S.R., Le, H.D., and Panda, S. (2009). Time of feeding and the intrinsic circadian clock drive rhythms in hepatic gene expression. Proc. Natl. Acad. Sci. USA *106*, 21453–21458.

Walton, K.M., Fisher, K., Rubitski, D., Marconi, M., Meng, Q.J., Sládek, M., Adams, J., Bass, M., Chandrasekaran, R., Butler, T., et al. (2009). Selective inhibition of casein kinase 1 epsilon minimally alters circadian clock period. J. Pharmacol. Exp. Ther. *330*, 430–439.

Westermark, P.O., and Herzel, H. (2013). Mechanism for 12 hr rhythm generation by the circadian clock. Cell Rep *3*, 1228–1238.

Young, M.W., and Kay, S.A. (2001). Time zones: a comparative genetics of circadian clocks. Nat. Rev. Genet. *2*, 702–715.

Yuan, Q., Metterville, D., Briscoe, A.D., and Reppert, S.M. (2007). Insect cryptochromes: gene duplication and loss define diverse ways to construct insect circadian clocks. Mol. Biol. Evol. *24*, 948–955.

Zhan, S., Merlin, C., Boore, J.L., and Reppert, S.M. (2011). The monarch butterfly genome yields insights into long-distance migration. Cell *147*, 1171–1185.

Zhang, L., Hastings, M.H., Green, E.W., Tauber, E., Sladek, M., Webster, S.G., Kyriacou, C., and Wilcockson, D. (2013). Dissociation of circadian and circatidal time-keeping in the marine crustacean *Eurydice pulchra*. Curr. Biol. *23*. Published online September 26, 2013. http://dx.doi.org/10.1016/j.cub.2013.08.038.

Zhu, H., Yuan, Q., Briscoe, A.D., Froy, O., Casselman, A., and Reppert, S.M. (2005). The two CRYs of the butterfly. Curr. Biol. *15*, R953–R954.

Zhu, H., Sauman, I., Yuan, Q., Casselman, A., Emery-Le, M., Emery, P., and Reppert, S.M. (2008). Cryptochromes define a novel circadian clock mechanism in monarch butterflies that may underlie sun compass navigation. PLoS Biol. *6*, e4.

Supplemental Information

EXTENDED EXPERIMENTAL PROCEDURES

Worm Culture and Light Conditions

For circadian and circalunar experiments, worms were housed in separated shelving systems using LED lights at 8000K. Nocturnal light intensities mimicking "full moon" were between 1-10lux according to previously published conditions (Hauenschild, 1960). Animals were exposed to a circadian light regime of 16 hr light and 8 hr darkness. Daylight was switched on at ZT0 and switched of at ZT16. NM and FR-FM: no light from ZT16 to ZT24; FM: dim nocturnal light from ZT16 to ZT24.

Gene Identification

Fragments of *Pdu-pdp1, Pdu-vrille*, *Pdu-timeless*, *Pdu-ck*1δ/ε sequences and full fragments of *Pdu-clock,Pdu-bmal, Pdu-tr-cry and Pdu-4-6-photolyase* sequences were identified based on BAC and EST sequences. *Pdu-clock:* upper primer: 5′ TGTGGTCTA TAATGGCTTGGATT 3′, lower primer: 5′-AAACGTCAGAACTGTGATTAGGC-3′ Tm: 53°C (full sequence).

Pdu-bmal: upper primer: 5′-ATGGGTCGGACTCCGATCGTTTCG 3′, lower primer: 5′ CTATAACGGCCAAGGAAAGTCAG-3′ Tm: 61°C (full sequence).

Pdu-tr-cry: upper primer: 5′-GGGTGGATAATCAGGCTTGTATTTCAAC 3′, lower primer: 5′ GTATCAATCGGTTTCTGTGACTC 3′ Tm: 58°C (full sequence).

Pdu-4-6-photolyase: upper primer: 5′ TTGCACAGCTTTGCAGAGAATCCAC 3′, lower primer: 5′ GATCTAATAACAACAATGAACA CATAG 3′ Tm: 58°C (full sequence).

Pdu-pdp1: upper primer: 5′ ACATTATGACGTGTAAGTATTTATGC 3′, lower primer: 5′ ACACTCAATTCTGCAGGTACACC 3′ Tm: 55°C.

Pdu-vrille: upper primer: 5′ ATGGACCCCGCTTGCAGAACGAACAC 3′, lower primer: 5′CTCCGGCTCATCCGGGTGCTTC 5′ Tm: 63°C.

Pdu-timeless: upper primer: 5′ TTGGTCTACTTGGACGAGGAAGAAG 3′, lower primer: 5′ CCCTAGGCAGATTTATGCAAGTCTC 3′ Tm: 58°C.

*Pdu-ck*1δ/ε: upper primer: 5′ GAATTCCCGGGATGTAAATAATGGCG 3′, lower primer: 5′ TAGGCCTCGGAGTTGGTCATGACAC 3′ Tm: 61°C.

Fragments of all other genes were first identified by degenerated PCR, and (if necessary) expanded by RACE PCR using QIAGEN Hot Star Taq (203205). Primers were used at 3μM.

Degenerated PCR program:

15min 95°C /5 cycles with 1min 94°C, 2min Tm −5°C, 1min 94°C / 35 cycles with 1min 94°C, 2min Tm, 4min 72°C/ final elongation of 10min 72°C.

Pdu-L-cry: upper primer: 5′GAYGGNGARGTIGCIGGIAC 3′, lower primer: 5′ACCCACATCCARTTICCIGCRCAIAC 3′ Tm:49°C.

Pdu-timeout: upper primer: 5′ YTNHTNGTXAAYYTXACXCARCC 3′, lower primer: 5′ YTCNARCATYTTXAVRAAXAVRTG 3′ and nested upper primer: 5′ GAYGAYGCXWSXHTXCAYGAYCA 3′, Tm: 48°C; nested lower primer: 5′ NGTNADCATCATYTCRTARTA 3′, Tm: 41°C.

Pdu-period: upper primer: 5′ ATHYTNGGITAYCCIAARGAY 3′, lower primer: 5′ RTTNARYTGNARIGGISWISWRCA 3′ and nested upper primer: 5′ ATHYTNGGI TAYCCXAARGAY 3′, Tm: 45°C; nested lower primer: 5′ CRTTRTGNGCXCKCA TICKRTAIGG 3′, Tm: 44.3°C.

Pdu-period Race: The 3′-end of the coding region was obtained by rapid amplification of cDNA ends. 1st primer: 5′ AT CACCGTTCTTCGCCCTCAT 3′, 2nd (nested) primer: 5′ GAACGTCTGTCCAGGCATCT 3′, UPM of the Clontech kit was used as counter-primer.

Phylogenetic Analyses

NJ trees were generated using ClustalX software (Larkin et al., 2007), correcting for multiple substitutions and bootstrapped by 1000 repetitions. For ML analysis, consensus trees were calculated, using TREE-PUZZLE (Schmidt et al., 2002) (v5.3), from 100 trees generated with IQPNNI (Vinh and Von Haeseler, 2004) (v3.3.2) using the BLOSUM62 substitution matrix and 4 categories of gamma-distributed substitution rates.

Primers Used in RT-PCR

PCR primers were designed using the ProbeFinder v.2.45 (Roche). The amplification reactions (20μl) contained 2x SYBR Green Master Mix (Applied Biosystems), 0.5μM of each primer and 5μl of the cDNA template. Each sample was run in duplicate along with control reactions that did not include reverse transcriptase. Amplification thermal profile was for 10 min at 95°C (for 15 s at 95°C, for 1 min at 60°C) x 40 cycles.

Pdu-cdc5: upper primer: 5′ CCTATTGACATGGACGAAGATG 3′, lower primer: 5′ TTCCCTGTGTGTTCGCAAG 3′

Pdu-bmal: upper primer: 5′ tccgatttatctccacgagaa 3′, lower primer: 5′ tcc gtctttacaggcagc a 3′

Pdu-period: upper primer: 5′ GGTCAACATGAAGTCGTACAGG 3′, lower primer: 5′ CACTGGTTTTCGGCTCAA G 3′

Pdu-clock: upper primer: 5′ tgagcgtatgaaagtatacaatgttct 3′, lower primer: 5′ tgagctggttctcatccttttc 3′

Pdu-tr-cry: upper primer: 5′ TGTTCAAATTCCATGGGACA 3′, lower primer: 5′- TGT TTTAGCCTCAGCCCATT −3′
Pdu-L-cry: upper primer: 5′ AGGACCTTGATGACAGCTTGA 3′, lower primer: 5′ TCTTGTCACTCCCCATTCCT 3′
Pdu-pdp1: upper primer: 5′ GAAACTGCGTCTCGAAATGA 3′, lower primer: 5′ TCCTCCTTAACTTCCACAGTCTTC 3′
Pdu-vrille: upper primer: 5′ GAAAGAAACTTCTCCGCAAAGCA 3′, lower primer: 5′ CTCATCCGGGTGCTTCAT 3′
Pdu-timeless: upper primer: 5′ ATGTCGCAGGAGACGTCAA 3′, lower primer: 5′ AGACCTTCAAGATGGGCAAC 3′
Pdu-timeout: upper primer: 5′ TGATGCATGCTGTGAAGGAT 3′, lower primer: 5′ GCCCACAGGTAGTAGGTCTCA 3′

Mounting and Microscopy

Fluorescently stained animals were mounted in DABCO-glycerol. Pictures were taken on a Zeiss Axioplan2 microscope using 20X Plan-Neofluar objective (dry) or 40X Neofluar objective (oil immersion). Images were recorded with Zeiss AxioCam MRC 5 camera using AxioVison 4.8 software.

SUPPLEMENTAL REFERENCES

Larkin, M.A., Blackshields, G., Brown, N.P., Chenna, R., McGettigan, P.A., McWilliam, H., Valentin, F., Wallace, I.M., Wilm, A., Lopez, R., et al. (2007). Clustal W and Clustal X version 2.0. Bioinformatics *23*, 2947–2948.

Schmidt, H.A., Strimmer, K., Vingron, M., and von Haeseler, A. (2002). TREE-PUZZLE: maximum likelihood phylogenetic analysis using quartets and parallel computing. Bioinformatics *18*, 502–504.

Vinh, S., and Von Haeseler, A. (2004). IQPNNI: moving fast through tree space and stopping in time. Mol. Biol. Evol. *21*, 1565–1571.

Cell PRESS
Open ACCESS

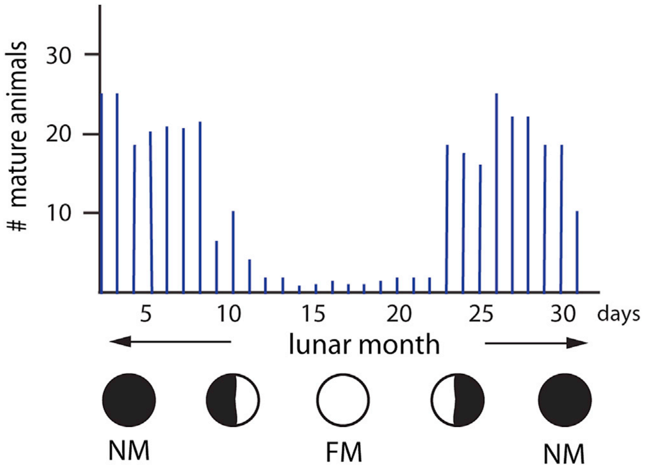

Figure S1. Synchronized Spawning Behavior of *Platynereis dumerilii*, Related to Figure 1
Numbers of mature animals oscillate in accordance with the lunar cycle. Figure redrawn from Hauenschild (1955).

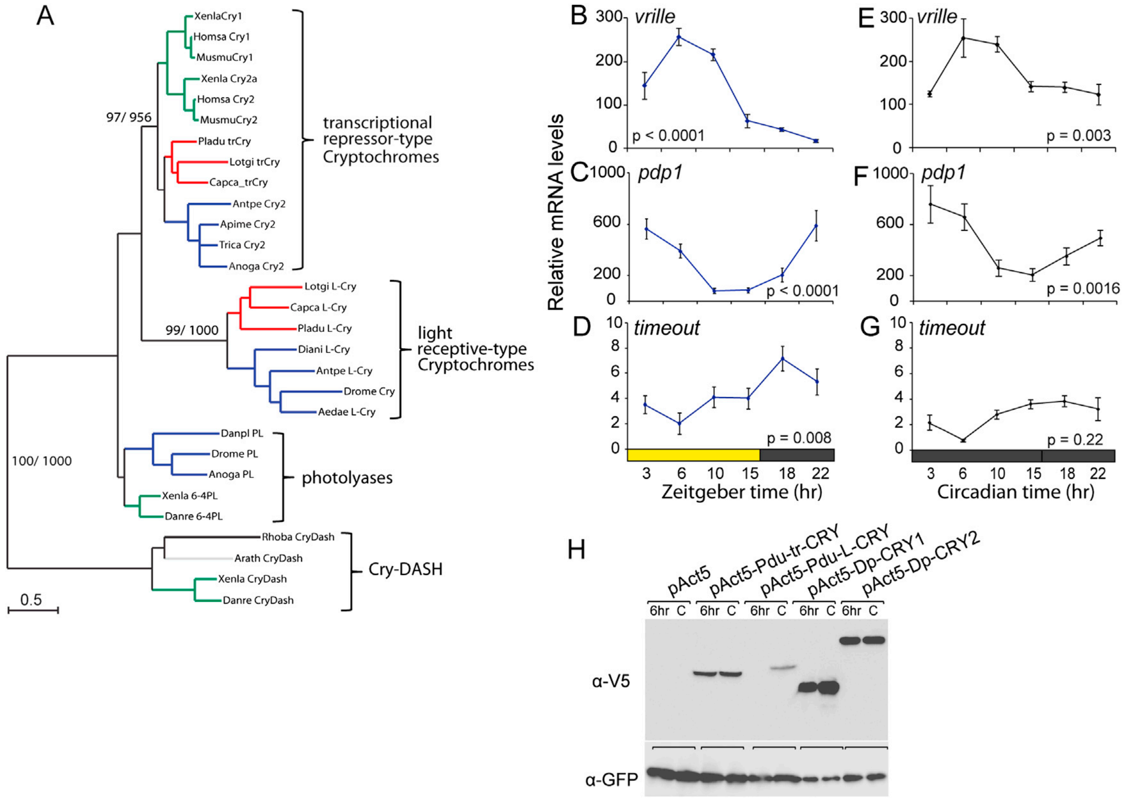

Figure S2. *Platynereis* L-Cry and tr-Cry Represent Members of the Light Receptive and Transcriptional Repressor-type Cryptochrome Groups, Respectively, Related to Figure 2

(A) *Platynereis* L-Cry and tr-Cry represent members of the light receptive and transcriptional repressor-type Cryptochrome groups, respectively. Maximum likelihood and neighbor joining trees group *Pdu*-L-Cry and *Pdu*-tr-Cry into the different classes with high branch support. The topology of the ML tree is shown, support values are given as ML/NJ at critical branches. Branch colors: green- vertebrates, red- lophotrochozoa, dark blue- insects, gray- plants, black- bacteria.

(B–G) Temporal profiles of clock gene RNA expression from *Platynereis* heads; (B-D) NM under circadian light regime (E-G) NM under constant darkness. n = 5-15 (B-D); n = 3-6 (E-G); 4-5 heads/n. Values are means ± SEM p value determined by one-way ANOVA.

(H) Example of one of the western blots used for the quantification shown in (Figure 2L). 6h: 6hr light puls; c: dark control. Related to Figure 2.

Cell PRESS Open ACCESS

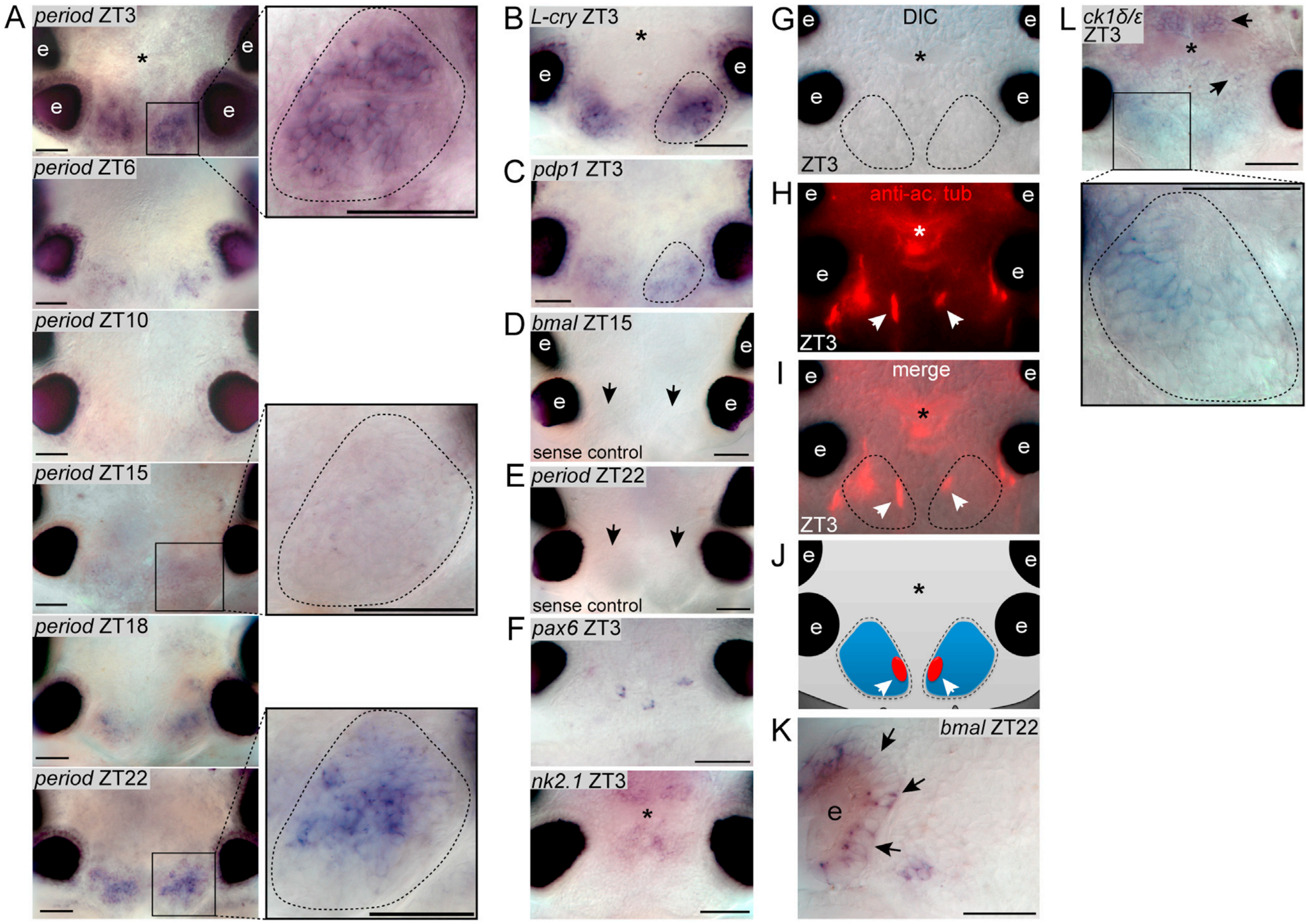

Figure S3. Specific Expression of *Platynereis* Circadian Clock Gene Orthologs in the Medial Forebrain, Related to Figure 3

(A–C) Whole mount in situ hybridization (WMISH) of *period*, *L-cry* and *pdp1* on premature adult *Platynereis* heads. Timeline for *period* expression anti-correlates with *Pdu-bmal* expression timeline (see Figure 3A). Dotted oval outlines circadian clock gene expressing, oval-shaped nucleus. Dorsal views, anterior up. Compare to Figures 3A–3E.

(D and E) WMISHs of *period* and *bmal* sense controls. Arrows point at oval-shaped brain nucleus expressing circadian clock genes.

(F) WMISHs of non-circadian transcription factors *pax6* and *nk2.1* show that the co-expression of circadian clock genes in the oval-shaped nucleus is specific.

(G–J) Co-localization of circadian clock gene expression with ciliary photoreceptor cells. Arrows point at the large cilia of the ciliary photoreceptors, counter-stained by the anti-acetylated tubulin antibody, visualizing stabilized microtubules (Arendt et al., 2004).

(G) DIC image.

(H) Fluorescent image visualizing the anti-acetylated tubulin antibody staining (red).

(I) Overlay of N and O. (J) scheme of P. (K) Expression of *bmal* in cells of the adult eye as revealed by WMISH on a *Platynereis* eye color mutant. (L) WMISH of *ck1 delta/epsilon* shows co-expression with *Platynereis* circadian clock genes in the oval-shaped nucleus (see magnified view). In addition *ck1 delta/epsilon* expressing cells are located anterior and posterior to the neuropil (arrow). Dorsal views, anterior up, asterisk- position of major brain neuropil. e- adult eye. Scale bar 50μm. Related to Figure 3.

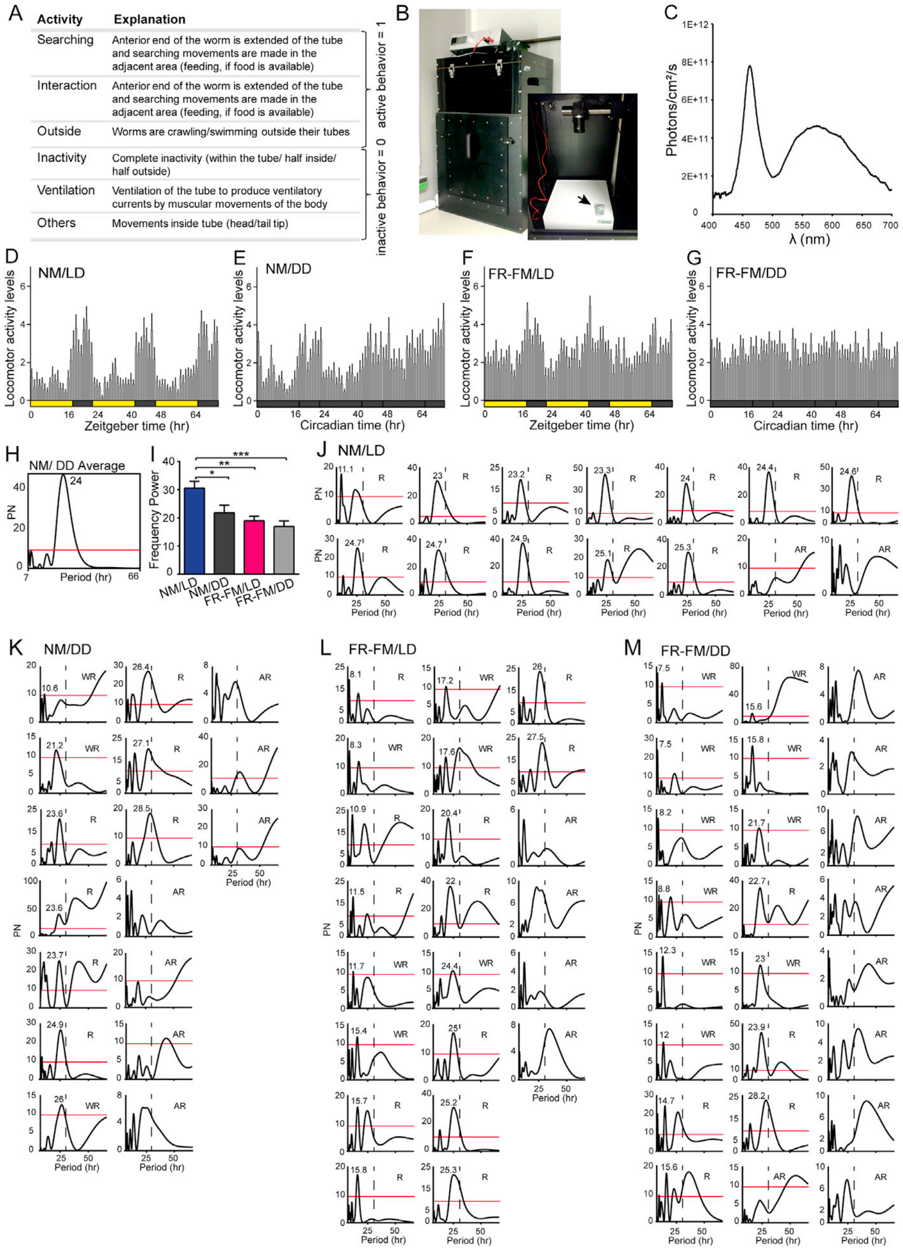

Figure S4. *Platynereis* Circadian Locomotor Activity under Laboratory Conditions, Related to Figure 4

(A) Types of activities scored as either active (1) or inactive (0) for analyses of *Platynereis* circadian locomotor behavior.

(B) Recording set-up closed (large image), open (small image) showing LED infrared array (white box), camera and the control recording device for light and temperature (= HOBO temperature tight 28000DP logger), arrow).

(C) Spectral analysis of white light LED's (OSRAM 24V) used in the black box set up (visible light spectrum).

(D–G) Mean locomotor activity (hourly average ± SEM) over 3 consecutive days containing rhythmic and arrhythmic animals, (D) N = 14, (E) N = 17, (F) N = 22, (G) N = 24. Compare to Figures 4A and 4F–4H.

(H) Average periodogram for NM/DD conditions using Lomp-Scargle analysis. NM/DD: worms were entrained normally with circadian and circalunar illumination conditions. Recordings were performed during NM in complete darkness. Compare to Figures 4F, 4J, and 4L.

(I) Frequency power was calculated for all rhythmic animals. Worms under NM LD/DD versus FR-FM LD/DD show significant differences in strength of the rhythm. Error bars represent ±SEM. Student's t test. *p < 0.05, **p < 0.01, ***p < 0.001.

(J–M) Individual Lomp-Scargle periodograms arranged according to increased period length for (J) NM/LD, (K) NM/DD, (L) FR-FM/LD, (M) FR-FM/DD, see D–G for illumination. Red line indicates significant p-level = 0.05. Period length > 29 were excluded from the 3 day analysis (indicated by dotted line) R; rhythmic, WR; weakly rhythmic, AR; arrhythmic. Related to Figure 4.

Cell PRESS
Open ACCESS

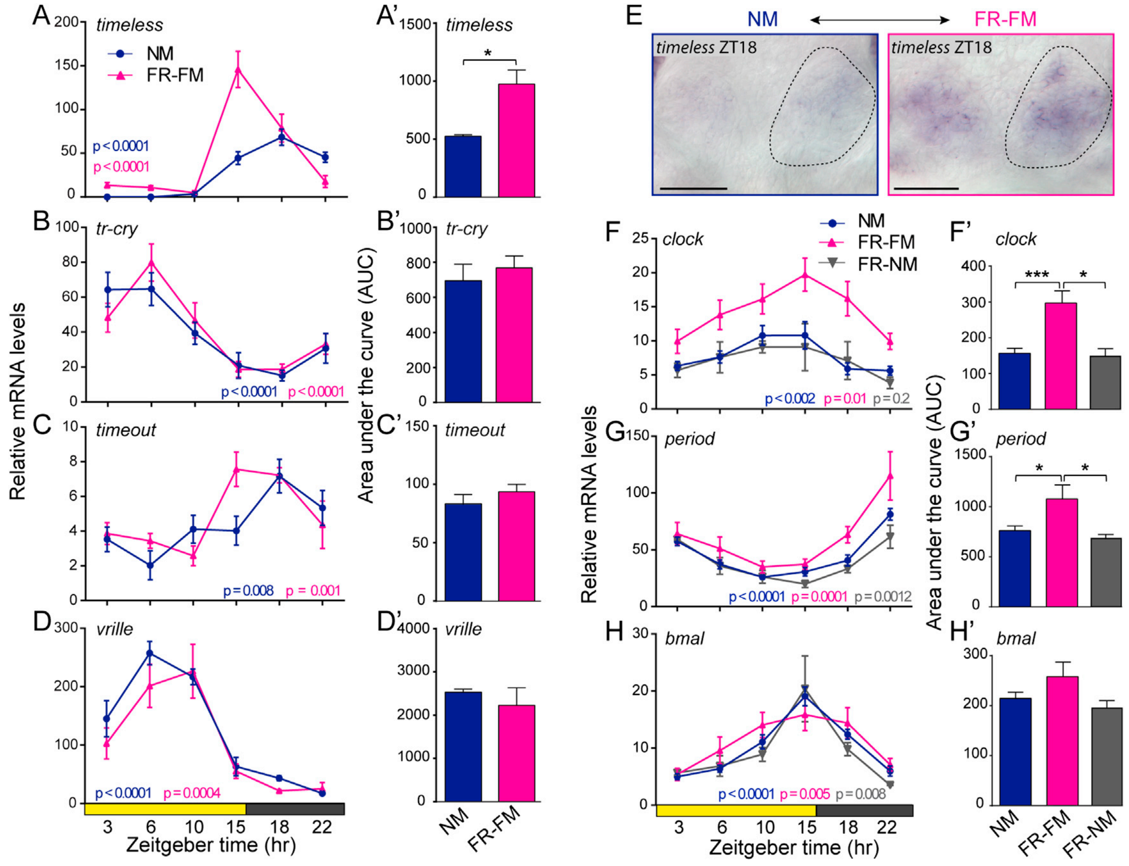

Figure S5. Circadian Transcriptional Regulation of *timeless* Is under Circalunar Clock Control, Related to Figure 5

(A–D) Temporal profiles of clock gene RNA expression measured by qPCR under NM and FR-FM conditions from *Platynereis* heads. See Figure 1C for detailed information on circalunar-light regime. Values are means ± SEM, NM n = 5-15, FR-FM n = 3-11; 4-5 heads/n. p-value determined by one-way ANOVA.

(A′–D′) Overall daily transcript levels calculated as area under the curve (AUC) based on 24h expression data shown in (A–D). Significant differences by Wilcoxon signed rank test. *p < 0.05.

(E) Expression of *timeless* increases in the circadian core brain domains in FR-FM compared to NM. Scale bar 50µm. Oval- outline of circadian clock brain area (also compare to Figures 3A–3E).

(F–H) Temporal profiles of clock gene mRNA expression under FR/NM (gray graph) resemble that of NM and FR-FM. Values are means ± SEM, FR-NM n = 3. Data for NM and FR-FM re-plotted from Figures 5B–5D.

(F′–H′) Area under the curve (AUC) based on 24h data shown in (F–H). *Platynereis* overall expression dynamics of *clock* and *period* are significantly decreased in FR-NM (gray bar) compared to FR-FM and are similar to NM (FR-NM n = 3; 4-5 heads/n). Data for NM and FR-FM re-plotted from Figure 5B'-D'. Significant differences by Wilcoxon signed rank test. *p < 0.05, ***p < 0.001. Related to Figure 5.

Cell
PRESS
Open
ACCESS

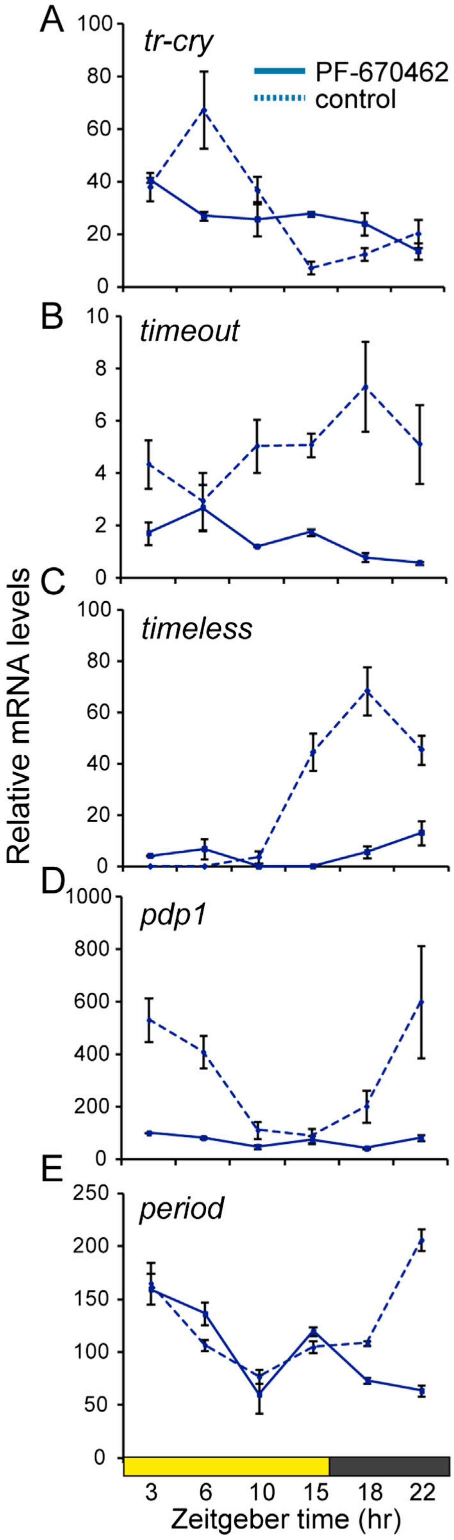

Figure S6. PF-670462 Treatment Disrupts Circadian Transcript Oscillations, Related to Figure 6

(A–E) Clock gene transcriptional oscillations are severely affected under PF-670462 treatment compared to non-treated controls (dashed line). Values are means ± SEM; n = 3; 4-5 heads/n. Related to Figure 6.
